# The energy landscape of *N*-ribosidic bond cleavage catalysed by 2′-deoxynucleoside 5′-phosphate *N*-hydrolase 1

**DOI:** 10.1042/BCJ20253400

**Published:** 2025-12-17

**Authors:** Anna E. Carberry, Tamal Das, David J. Harrison, Jennifer S. Hirschi, Rafael G. da Silva

**Affiliations:** 1 School of Biology, Biomedical Sciences Research Complex, University of St Andrews, St Andrews, KY16 9ST, U.K.; 2 Department of Chemistry, Binghamton University, Vestal, NY 13850, U.S.A.; 3 School of Medicine, University of St Andrews, St Andrews, KY16 9TF, U.K.; 4 NuCana plc, Edinburgh, EH12 9DT, U.K.

**Keywords:** 2′-deoxynucleoside 5′-phosphate*N-*hydrolase 1, covalent catalysis, DNPH1, QM/MM calculations, rate-limiting step

## Abstract

The human enzyme 2′-deoxynucleoside 5′-phosphate *N*-hydrolase 1 (*Hs*DNPH1) catalyses the *N*-ribosidic bond hydrolysis of the non-canonical nucleotide 5-hydroxymethyl-2′-deoxyuridine 5′-monophosphate (5hmdUMP), producing 5-hydroxymethyluracil and 2-deoxyribose 5-phosphate, preventing 5hmdUMP incorporation into DNA. This reaction is unusual for a nucleoside/nucleotide *N*-hydrolase as it proceeds by a double-displacement mechanism whereby Glu104 nucleophilically attacks 5hmdUMP to form a covalent 5-phospho-2-deoxyribosylated enzyme intermediate, which is subsequently hydrolysed. Here, we used site-directed mutagenesis and UV-VIS differential spectroscopy to show a shift in the 5hmdUMP absorbance spectrum upon binding to *Hs*DNPH1 before *N*-ribosidic bond cleavage. This spectral shift can be monitored independently in different wavelengths to characterise the kinetics of *Hs*DNPH1-5hmdUMP binary complex formation. The one-step binding mechanism produces a calculated equilibrium dissociation constant in agreement with that obtained by isothermal titration calorimetry. Pre-steady-state kinetics under multipleturnover conditions revealed absence of a burst of substrate consumption at a wavelength where binding does not lead to change in absorbance. This indicates steps after *N*-ribosidic bond cleavage are fast. Single-turnover kinetics, where the signal comes solely from the first half-reaction, indicate *N*-ribosidic bond cleavage in the first half-reaction is rate-determining for *k*_cat_. Linear free energy relationships between leaving groups with increased p*K*_a_ and *k*_cat_/*K*_M_ suggest a late transition state with significant negative charge accumulation in the leaving group during *N*-ribosidic bond cleavage. These results were complemented by on-enzyme QM/MM calculations of the first half-reaction to reveal an anionic leaving group in an S_N_2-like transition state with C1′–N1 bond cleavage more advanced than C1–O bond formation with Glu104.

## Introduction

Breast cancer susceptibility genes 1 and 2 (*BRCA1* and *BRCA2*) are tumour suppressor genes whose products are essential for homologous recombination (HR)-mediated DNA double-strand break repair [1]. Germline mutations in *BRCA1* and/or *BRCA2* (present in approximately 1 in 400 people) render individuals at significant risk of developing breast, prostate, pancreatic and ovarian cancers, often associated with poor prognosis [2]. These cancer cells are dependent on poly(ADP)-ribose polymerase 1 (PARP1) for single-strand DNA repair; thus, PARP inhibitors (PARPi) were developed specifically to treat *BRCA^-/-^* cancers by trapping PARP1 at single-strand breaks in DNA, causing replication fork collapse and cytotoxicity to the rapidly proliferating cancer cells [3]. However, in the clinic, *BRCA^-/-^* tumours often acquire resistance to PARPi [4].

Human 2′-deoxynucleoside 5′-phosphate *N-*hydrolase 1 (*Hs*DNPH1) (EC 3.2.2.-) was recently identified as a promising target for inhibition to re-sensitise resistant *BRCA^-/-^* cancer cells to PARPi [4]. *Hs*DNPH1 catalyses the *N*-ribosidic bond cleavage of cytotoxic 5-hydroxymethyl-2′-deoxyuridine 5′-monophosphate (5hmdUMP) to produce 5-hydroxymethyluracil (5hmUra) and 2-deoxyribose 5-phosphate. In the absence of *Hs*DNPH1 catalytic activity, 5hmdUMP accumulates in the nucleotide pool and is erroneously incorporated into DNA, triggering 5hmUra removal and PARP1 recruitment to the abasic site, where it can be trapped by PARPi [4].

**Figure 1:**
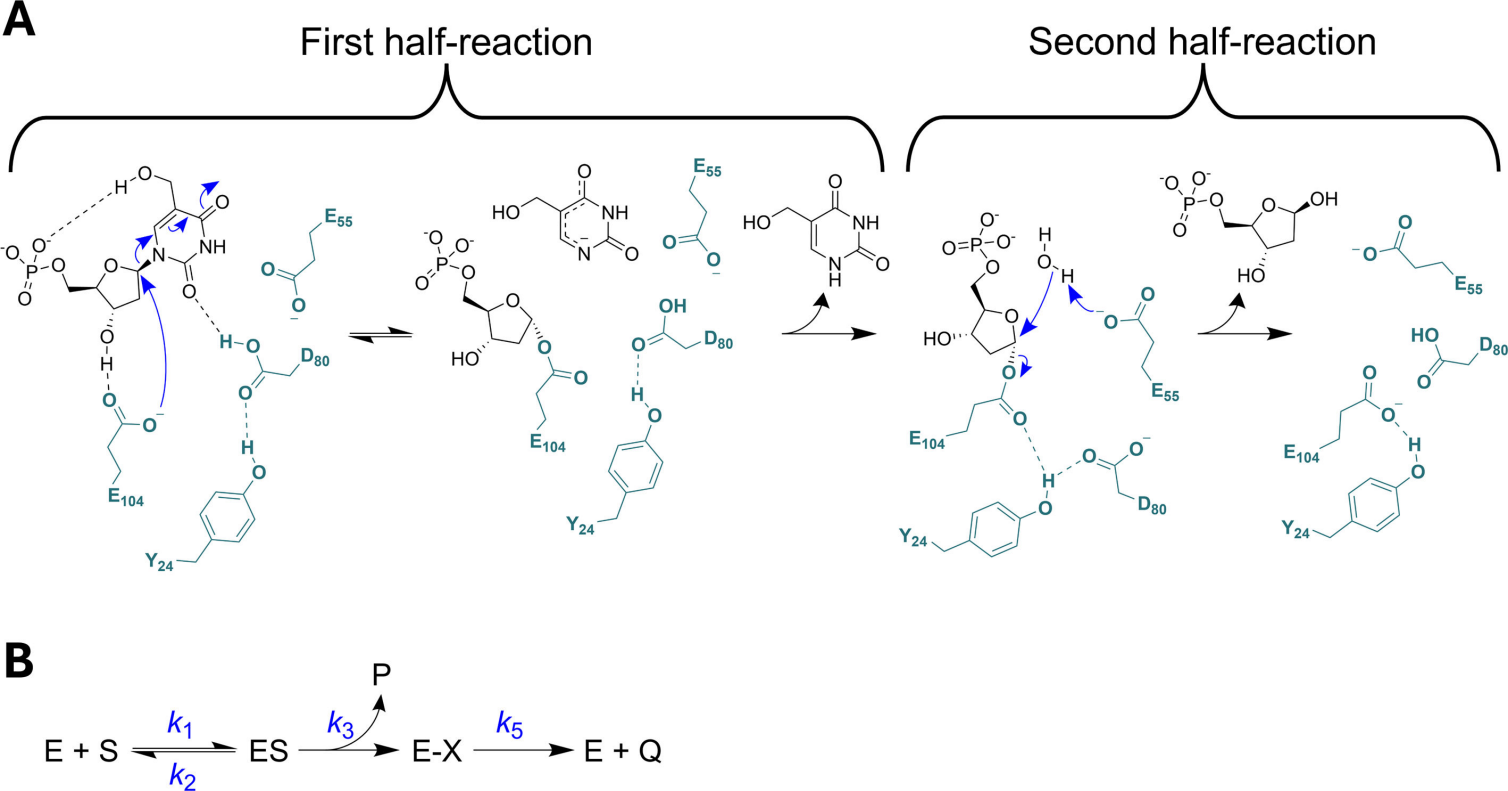
Double-displacement mechanism proposed for *Hs*DNPH1 catalysis. **(A**) Catalysis at the active site. Substrate and products are depicted in black, whereas active-site residues, in teal. (**B**) The minimum kinetic sequence for the *Hs*DNPH1-catalysed reaction. Substrate binding leads to formation of the Michaelis complex (ES) which either dissociates to free enzyme or proceeds irreversibly to form the covalent intermediate (**E–X**) and release the first product. E–X is then hydrolysed to the final product and free enzyme. For simplicity, *k*_3_ and *k*_5_ each encompasses chemistry and product release.

To help inform inhibitor design, recent studies have focused on elucidating the *Hs*DNPH1 catalytic mechanism using protein crystallography, site-directed mutagenesis, assay development, enzyme kinetics and transition-state analogues [5–9]. While most (2′-deoxy)nucleoside *N*-hydrolases catalyse a direct nucleophilic attack of activated water on C1′ of the nucleoside with inversion of configuration at the anomeric carbon [10], *Hs*DNPH1 and its rat orthologue operate by covalent catalysis in a double-displacement mechanism with retention of configuration on the anomeric carbon [5,6,11] (Figure 1A). In the first half-reaction, catalysis relies on an intricate network of hydrogen bonds (H-bonds) involving the substrate and the catalytic triad Y24-D80-E104, with nucleophilic attack of E104 on 5hmdUMP C1′ to displace a possibly anionic 5hmUra and form a 5-phospho-2-deoxyribosylated enzyme intermediate, as surmised from crystal structures [6,7]. Based on pH-rate profiles and site-directed mutagenesis, D80 is likely the general acid involved in the protonation of the anionic 5hmUra to the neutral 5hmUra product [7]. In the second half-reaction, water is activated by E55 via general-base catalysis for nucleophilic attack on the covalent intermediate to produce the 5-phospho-2-deoxyribose product and regenerate the enzyme. This step is slowed down in the E55Q-*Hs*DNPH1 variant, allowing the covalently modified enzyme to be trapped and its crystal structure solved [6]. These steps are corroborated by crystal structures with analogues of the presumed transition state of each half-reaction [8].

While there is agreement on qualitative aspects of the reaction such as the nature of the covalent intermediate and the identity and likely role of the key catalytic residues, conflicting lines of evidence exist regarding the kinetics of the catalysed reaction. With rat DNPH1, a close orthologue of *Hs*DNPH1 [12], results from rapid-quenching methods suggested the steady-state catalytic constant (*k*_cat_) is limited by the first half-reaction, with the caveat that the non-physiological, slow-reacting substrate 2′-deoxyguanosine 5′-monophosphate was used [11]. In contrast, with *Hs*DNPH1 and 5hmdUMP, the second half-reaction was proposed as rate-limiting for *k*_cat_, based on protein fluorescence measurements in a stopped-flow spectrofluorimeter [6]. Figure 1B depicts the minimum kinetic sequence for *Hs*DNPH1.

In the present work, we employed differential UV-VIS absorbance, UV-VIS-based stopped-flow spectrophotometry, site-directed mutagenesis, isothermal titration calorimetry (ITC) and solvent deuterium isotope effects to resolve the rate-limiting step of *Hs*DNPH1 catalysis. Furthermore, we used linear free energy relationships coupled with quantum mechanics/molecular mechanics (QM/MM) calculations of the first half-reaction to reveal the nature of the 5hmUra leaving group at the transition state and provide an atomistic model of catalysis by *Hs*DNPH1.

## Results

### **Time-dependent UV spectra of** the ***Hs*DNPH1 reaction reveal isosbestic points**

**Figure 2:**
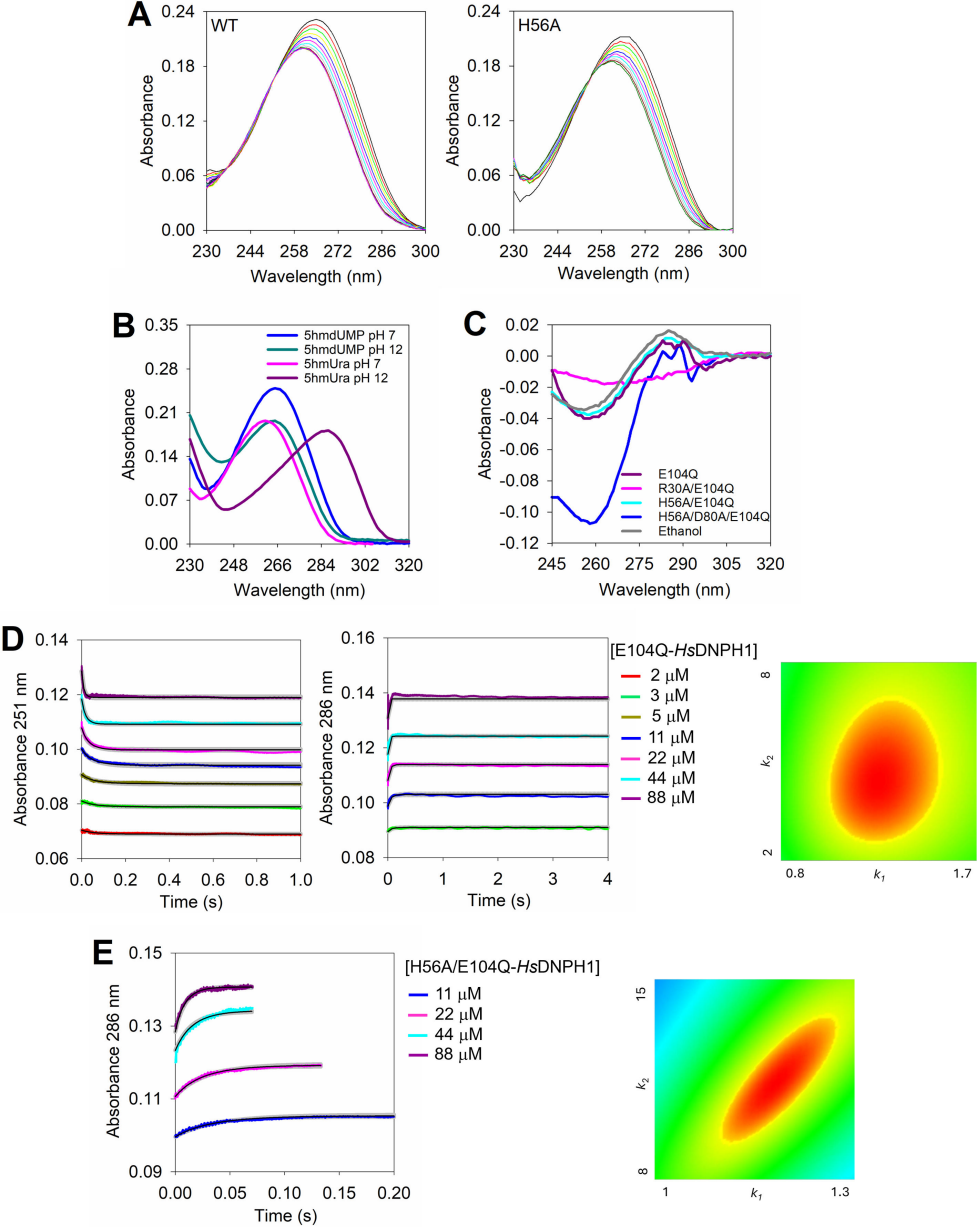
Time-dependent UV spectra and binding kinetics studies with *Hs*DNPH1 at 25°C and pH 7.0. (**A**) Time-dependent UV spectra of the WT- and H56A-*Hs*DNPH1-catalysed reactions. The region in the spectra between 230 nm and 244 nm was not included in the reaction analysis of the H56A variant because it also changes in the control lacking enzyme at the long timescale needed with this variant (Supplementary Figure S1). (**B**) UV spectra of 5hmdUMP and 5hmUra at neutral and basic pH. (**C**) Differential UV spectra of 5hmdUMP bound to *Hs*DNPH1 variants. (**D**) Binding of E104Q-*Hs*DNPH1 to 5hmdUMP and FitSpace contour plot of the best-fit model relative to the data. (**E**) Binding of H56A/E104Q-*Hs*DNPH1 to 5hmdUMP and FitSpace contour plot of the best-fit model relative to the data. In (**D**) and (**E**), lines in colour are experimental data, black lines are numerical integration-based fit to a single-step binding model, and grey lines are boundaries produced by FitSpace analysis. In the contour plots, constrained boundaries are defined by regions in red; *k*_1_ is in units of μM^-1^ s^-1^, and *k*_2_, in s^-1^.

Time-dependent UV absorbance spectra were collected for the reaction catalysed by WT-*Hs*DNPH1 and H56A-*Hs*DNPH1 (H56 is modestly important for catalysis [6,7]) (Figure 2A). Both WT- and H56A-*Hs*DNPH1 produced spectra with an isosbestic point at ~251 nm; however, the spectra for the reaction with WT-*Hs*DNPH1 also revealed an isosbestic point at ~237 nm. Supplementary Figure S1 shows a close-up view of the isosbestic points with each enzyme variant, revealing that the isosbestic point ~251 nm is marginally displaced to ~254 nm with H56A-*Hs*DNPH1. However, it should be pointed out that the time-dependent spectra with H56A-*Hs*DNPH1 were collected over 1.5 h, and there is significant enzyme-independent change between 230 nm and 244 nm, and modest change near 254 nm, of the 5hmdUMP spectrum during that time (Supplementary Figure S1). This is also the likely reason why the isosbestic point at ~237 nm is not present with this variant. An isosbestic point defines a wavelength in the time-dependent spectra where two or more species have the same extinction coefficient, such that the absorbance at that wavelength does not change. While a single isosbestic point is associated with the absence of a reaction intermediate [13], an additional isosbestic point often suggests the presence of an intermediate [14].

The UV spectra of 5hmdUMP and 5hmUra in the absence of enzyme were also collected at pH 7.0 and 12.0 (Figure 2B). These pHs were chosen because *Hs*DNPH1 shows maximum *k*_cat_ at pH 7.0 [7], and 5hmUra has a p*K*_a_ of 9.5, above which it is deprotonated to form an anionic nucleobase [15] (Supplementary Figure S2). In accordance with the literature, 5hmdUMP has an absorbance maximum at 264 nm [15], although the intensity is lower at pH 12.0, while the absorbance of 5hmUra at 264 nm at pH 7.0 decreases, and its maximum shifts to 261 nm [7,15]. Very similar absorbance intensities are observed for 5hmdUMP (pHs 7.0 and 12.0) and 5hmUra (pH 7.0) near 251 nm, in agreement with an isosbestic point for the hydrolytic reaction at that wavelength. At pH 12.0, the 5hmUra absorbance maximum is red-shifted to 286 nm, as previously reported [15], and absorbance decreases around 251 nm.

### Binding to *Hs*DNPH1 shifts the UV absorbance of 5hmdUMP

As a proxy for WT-*Hs*DNPH1 to evaluate substrate binding preceding chemistry, the E104Q-*Hs*DNPH1 was produced (Supplementary Figures S3 and S4), as replacement of the nucleophilic E104 by glutamine was shown to render *Hs*DNPH1 catalytically inactive, but still able to bind the substrate [6]. HPLC analysis of the reaction in the presence of E104Q-*Hs*DNPH1 confirmed the lack of catalytic activity (Supplementary Figure S5). Surprisingly, differential UV spectroscopy, where the spectra of free enzyme and free 5hmdUMP are subtracted from the spectra of the mixture of enzyme and 5hmdUMP (Figure 2C) showed that formation of E104Q-*Hs*DNPH1-5hmdUMP binary complex results in a decrease in absorbance around 251 nm and an increase in absorbance around 286 nm, reminiscent of the spectrum of the anionic 5hmUra, even though the *N*-ribosidic bond is still intact.

To check the effect of replacing catalytic residues other than E104 [7] on the UV spectrum of 5hmdUMP upon enzyme binding, additional mutations were introduced to the background of the E104Q substitution to produce H56A/E104Q-*Hs*DNPH1, R30A/E104Q-*Hs*DNPH1, and H56A/D80A/E104Q-*Hs*DNPH1 (Supplementary Figures S3 and S4). The differential UV spectrum of the H56A/E104Q-*Hs*DNPH1-5hmdUMP complex is very similar to that of the E104Q-*Hs*DNPH1-5hmdUMP, while formation of the R30A/E104Q-*Hs*DNPH1-5hmdUMP complex leads to loss of most of the differential spectrum features. The H56A/D80A/E104Q-*Hs*DNPH1-5hmdUMP complex formation abolishes the change in absorbance around 286 nm, but it causes a more pronounced decrease in absorbance around 251 nm (Figure 2C). Interestingly, the differential spectrum of 5hmdUMP in ethanol (dielectric constant of ~24) is similar to that of 5hmdUMP in complex with E104Q-*Hs*DNPH1 (Figure 1C).

### *Hs*DNPH1 binds 5hmdUMP in a single step

Taking advantage of the change in absorbance upon binary complex formation, the kinetics of E104Q-*Hs*DNPH1 binding to 5hmdUMP was monitored at 251 nm and 286 nm in independent rapid mixing experiments, yielding the predicted time-dependent decrease and increase in absorbance, respectively (Figure 2D). Global fit of both experiments simultaneously to a single-step binding model (Supplementary Figure S6) via numerical integration as implemented in KinTek Global Explorer [16] yielded reasonably constrained rate constants (Supplementary Table S1), as judged by FitSpace contour analysis [17] (Figure 2D). A similar experiment with H56A/E104Q-*Hs*DNPH1 monitored at 286 nm (Figure 2E) showed a modest effect of the H56A substitution on substrate binding (Supplementary Table S1). In the latter experiment, the elongated FitSpace plot along the *k*_2_ axis reveals that the ratio *k*_2_/*k*_1_ is better defined than the dissociation rate constant alone [17,18]. The key observation here is that the fast absorbance changes at 251 nm and 286 nm are attributed to substrate binding.

**Figure 3:**
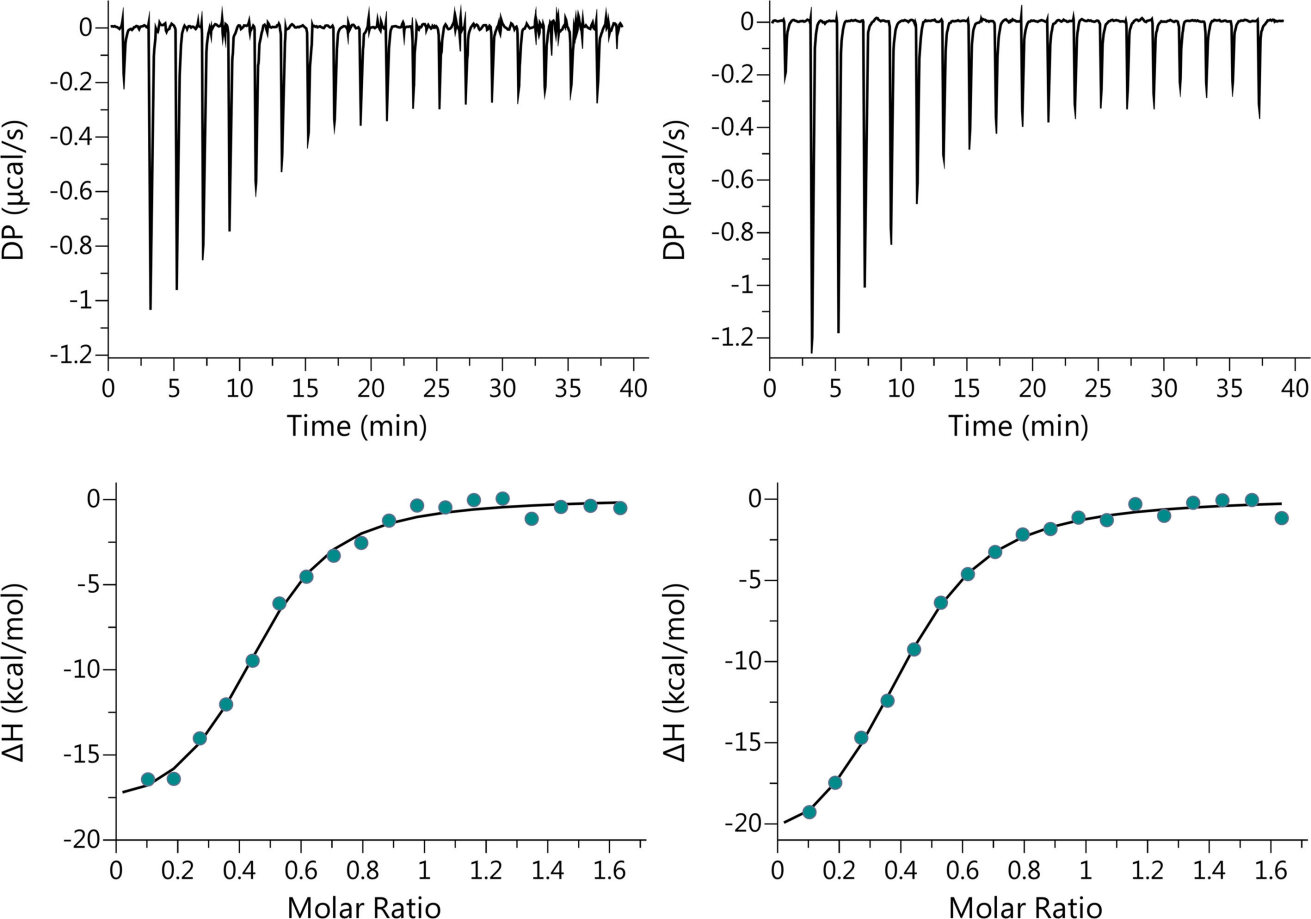
Equilibrium thermodynamics of 5hmdUMP binding to E104Q-*Hs*DNPH1 by ITC. Two independent experiments were carried out at 25°C and pH 7.0. The data were best fitted to a single-site binding model with 2:1 (protein:ligand) stoichiometry. Values reported in the text are mean and where relevant, mean ± propagated fitting error, of two independent experiments.

The E104Q-*Hs*DNPH1 apparent equilibrium dissociation constant (*K*_D_^app^) for 5hmdUMP, calculated from the *k*_2_/*k*_1_ ratio, was 3.50 μM Supplementary Table S1. This is in reasonable agreement with the *K*_D_ of 1.6 ± 0.4 μM obtained from equilibrium binding by ITC (Figure 3), lending support to the one-step binding model. ITC also revealed that binding of E104Q-*Hs*DNPH1 to 5hmdUMP, with a Gibbs free energy (ΔG) of −7.9 kcal/mol, is enthalpically driven with ΔH of −21 ± 1 kcal/mol, which compensates for an unfavourable binding entropy (TΔS) of −13.3 kcal/mol. Intriguingly, the data could only be fitted with a model where ~0.42 ± 0.01 binding sites become occupied by 5hmdUMP. Since *Hs*DNPH1 is a dimer [5,6,8], this suggests the possibility that E104Q-*Hs*DNPH1 displays half-of-the-sites binding, where only one monomer of the dimer binds the substrate at any given time. In crystal structures of WT-*Hs*DNPH1 bound to dUMP and of D80N-*Hs*DNPH1 bound to 5hmdUMP, only one of the sites of the dimer in the asymmetric unit was occupied by the substrate [5,7]. However, since those Michaelis complex structures were obtained by soaking [5,7], possible crystal lattice constraints on substrate access to the other subunit cannot be ruled out.

### WT-*Hs*DNPH1 reaction does not display burst kinetics

**Figure 4:**
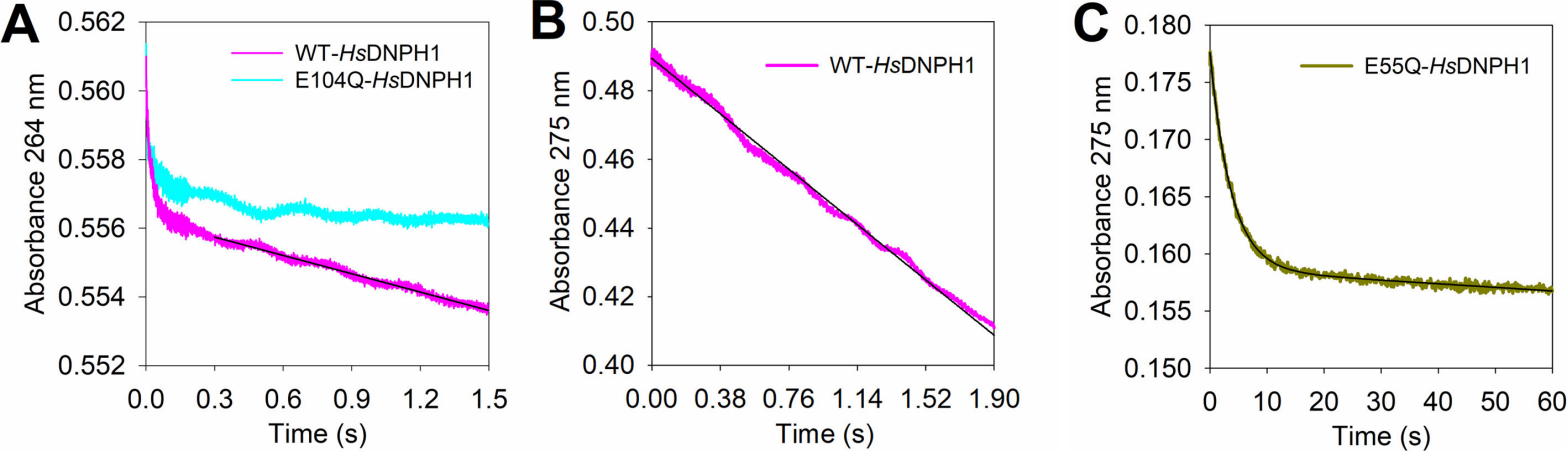
Rapid kinetics under multiple-turnover conditions at pH 7.0. (**A**) Reaction monitored at 264 nm. (**B**) and (**C**) Reactions monitored at 275 nm. Coloured lines represent data. Black lines in (**A**) and (**B**) are linear regressions of the data, while in (**C**), best fit of the data to equation 2.

To uncover the rate-limiting step of the reaction catalysed by WT-*Hs*DNPH1, pre-steady-state kinetics under multiple-turnover conditions were evaluated to search for a burst of on-enzyme *N*-ribosidic bond cleavage on the approach to the steady-state phase, which would indicate a step after the first half-reaction chemistry limits *k*_cat_ [19]. Initially, reactions monitored at 264 nm, a wavelength at which 5hmdUMP *N*-ribosidic bond cleavage leads to a decrease in absorbance [15] and at which the reaction has been previously assayed under steady-state conditions [7], showed what appeared to be a burst of *N*-ribosidic bond cleavage (Figure 4A), and the steady-state phase produced an apparent rate constant of 0.163 ± 0.001 s^-1^, close to the *k*_cat_ of 0.22 s^-1^ previously reported [7]. However, a control reaction under the same conditions but with WT-*Hs*DNPH1 replaced by the inactive E104Q-*Hs*DNPH1 produced a similar burst of absorbance decay, indicating the apparent burst phase was entirely due to substrate binding (Figure 4A), not chemistry. Interestingly, the similarity of the binding signal amplitude with E104Q-*Hs*DNPH1 and WT-*Hs*DNPH1 reinforces the hypothesis of half-of-the-sites binding in WT-*Hs*DNPH1. Table 1 summarises the wavelengths monitored here where absorbance changes upon binding and/or C1′−N1 bond cleavage.

**Table 1:** UV-VIS absorbance changes of 5hmdUMP with or without interaction with *Hs*DNPH1

Wavelength (nm)	Absorbance upon 5hmdUMP...	
	Binding to WT-*Hs*DNPH1	C1′–N1 bond cleavage
251	Decrease	Increase
264	Decrease	Decrease
275	Unaltered	Decrease
286	Increase	Decrease

The *k*_cat_ with WT- and H56A-*Hs*DNPH1 were measured at 275 nm (Supplementary Figure S7), a wavelength at which the absorbance difference between 5hmdUMP and 5hmUra is higher than at 264 nm (Figure 2A, Supplementary Figure S8). Best fit of the data to equation 1 yielded *k*_cat_ values (Supplementary Table S2) in agreement with those previously determined at 264 nm [7]. At 275 nm, no significant change in absorbance is observed upon substrate binding (Figure 2C), only upon *N*-ribosidic bond cleavage; thus, the pre-steady-state kinetics experiment under multiple-turnover conditions with WT-*Hs*DNPH1 was repeated at 275 nm, but this time no burst phase could be observed, only the steady-state phase (Figure 4B). Linear regression of the data produced an apparent rate constant of 0.230 ± 0.003 s^-1^, in close agreement with the *k*_cat_ of 0.21 s^-1^ determined at 275 nm (Supplementary Table S2). This suggests there is no rate-limiting step after on-enzyme *N*-ribosidic bond cleavage in the first half-reaction.

To probe further the robustness of this assay to reveal a true burst phase reflecting on-enzyme chemistry, the E55Q-*Hs*DNPH1 variant was produced (Supplementary Figures S3 and S4), as this substitution slows down hydrolysis of the covalent intermediate [6], but is not expected to have an effect on the first half-reaction, which should manifest as burst kinetics. This is exactly what is observed at 275 nm (Figure 4C), and best fit of the data to equation 2 yielded a *k*_burst_ of 0.270 ± 0.001 s^-1^, a burst phase amplitude of ~10 μM, the same concentration of E55Q-*Hs*DNPH1 used, and an apparent steady-state rate constant of 0.0018 ± 0.0001 s^-1^, in reasonable agreement with the E55Q-*Hs*DNPH1 *k*_cat_ obtained upon best fit of the data to equation 3 (Supplementary Figure S7, Supplementary Table S2), confirming a step after on-enzyme chemistry in the first half-reaction, presumably the chemistry of the second half-reaction, limits E55Q-*Hs*DNPH1 *k*_cat_. It also shows that for this variant, both active sites of the dimer are fully occupied by the substrate.

### Formation of 5hmUra is rate-determining for the WT-*Hs*DNPH1-catalysed reaction

The lack of burst kinetics with WT-*Hs*DNPH1 suggests the first half-reaction limits *k*_cat_. To test this hypothesis further and obtain information directly on the first half-reaction, pre-steady-state kinetics data under single-turnover conditions were collected to obtain a single-turnover rate constant (*k*_STO_). In these experiments, enzyme concentrations are in excess of substrate concentration [19], and monitoring the reaction at 251 nm and/or 286 nm may reveal the rates of formation and decay of enzyme-bound intermediates, perhaps even the putative anionic 5hmUra [5–7], provided it is sufficiently stable to accumulate during the reaction. In the case of WT-*Hs*DNPH1, the source of the absorbance change during the reaction ensures that the observed signal reflects only the first half-reaction, even though a full turnover is expected to take place. Furthermore, it is hypothesised that the absorbance will decrease at 251 nm and increase at 286 nm due to formation of the ES complex preceding chemistry and will increase and decrease afterwards in those wavelengths, respectively, owing to splitting of the *N*-ribosidic bond and formation of 5hmUra.

**Figure 5:**
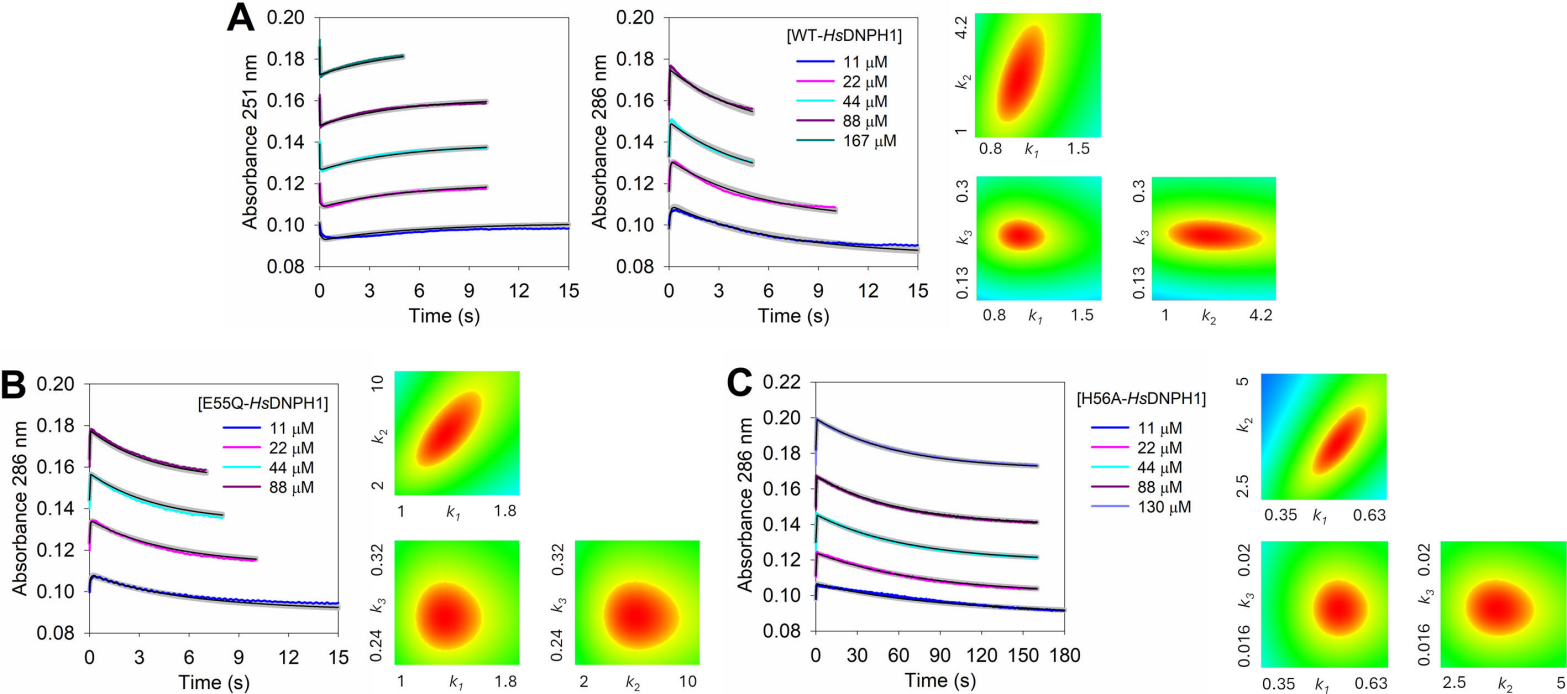
Single-turnover kinetics of *Hs*DNPH1 variants. (**A**) Single-turnover reaction of WT-*Hs*DNPH1 with 5hmdUMP and FitSpace contour plot of the best-fit model relative to the data. (**B**) Single-turnover reaction of E55Q-*Hs*DNPH1 with 5hmdUMP and FitSpace contour plot of the best-fit model relative to the data. (**C**) Single-turnover reaction of H56A-*Hs*DNPH1 with 5hmdUMP and FitSpace contour plot of the best-fit model relative to the data. Lines in colour are experimental data, black lines are numerical integration-based fit to a two-step reaction model, and grey lines are boundaries produced by FitSpace analysis. In the contour plots, constrained boundaries are defined by regions in red; *k*_1_ is in units of μM^-1^ s^-1^, and *k*_2_ and *k*_3_, in s^-1^.

This is what is observed when the single-turnover kinetics of WT-*Hs*DNPH1 is monitored independently at 251 nm and 286 nm (Figure 5A). Simultaneous fit of the whole dataset to the reaction sequence depicted in Supplementary Figure S9 resulted in rate constants displayed in Supplementary Table S2, well constrained by the two-step reaction model as judged by FitSpace analysis [17] (Figure 5A and Supplementary Table S2). The model in Supplementary Figure S9 starts with bimolecular binding to form the ES complex, with *k*_1_ and *k*_2_ in good agreement with the corresponding values from binding kinetics between E104Q-*Hs*DNPH1 and 5hmdUMP (Figure 2D and Supplementary Table S1), and the *k*_2_/*k*_1_ ratio of 2.36 μM close to that from E104Q-*Hs*DNPH1 binding kinetics (Figure 2D and Supplementary Table S1) and equilibrium binding by ITC (Figure 4). The second step is the *N*-ribosidic bond breaking step to form the covalent intermediate E-X (the 5-phospho-2-deoxyribosylated enzyme) and the first product P (5hmUra), governed by *k*_3_. At high enzyme concentrations, when all substrate is in the ES complex form and *k*_3_ is unimolecular, the single-turnover reaction is over in 5 s, consistent with *k*_cat_ of 0.21 s^-1^ (and consequent transit time of ~4.8 s for the overall reaction). This shows the consistency between data and reaction model.

Importantly, a *k*_3_ of 0.219 ± 0.001 s^-1^ is indistinguishable from *k*_cat_, indicating the overall reaction rate is determined by the rate of formation of 5hmUra and the covalent intermediate in the first half-reaction. Moreover, *k*_cat_/*K*_M_, the second order rate constant governing reaction at low substrate concentration, is described by equation 8 for the complete *Hs*DNPH1-catalysed reaction. Equation 8 also defines the flux through the first half-reaction described in Supplementary Figure S9. The steady-state data for WT-*Hs*DNPH1 in Supplementary Figure S7 did not allow an accurate measurement of *K*_M_ for 5hmdUMP, as its apparent value lies near 2.5 μM, the lowest substrate concentration possible with our assay. This places a lower limit on *k*_cat_/*K*_M_ of 8.4 × 10^4^ M^-1^ s^-1^ (Supplementary Table S2). Calculating the *k*_cat_/*K*_M_ using the rate constants obtained in the single-turnover experiment yielded a value of ~8.5 × 10^4^ M^-1^ s^-1^. These results indicate the kinetics of the first half-reaction are sufficient to describe the overall WT-*Hs*DNPH1 reaction kinetics at both low and high 5hmdUMP concentrations. In summary, coupled with the absence of burst kinetics, the single-turnover kinetics point to on-enzyme formation of 5hmUra in the first half-reaction as the rate-determining step for the WT-*Hs*DNPH1-catalysed reaction.


(Equation 8) $$\frac{k_{cat}}{K_{M}}=\frac{k_{1}k_{3}}{k_{2}+k_{3}}$$

To evaluate the effect on the first half-reaction of replacing catalytic residues, the single-turnover reactions of the E55Q- and H56A-*Hs*DNPH1 variants with 5hmdUMP were monitored at 286 nm (Figure 5B and C), and rate constants resulting from best fit to the model in Supplementary Figure S9 are shown in Supplementary Table S3. The E55Q substitution has a very modest effect on the first half-reaction, with a *k*_3_ indistinguishable from *k*_burst_ and comparable with the WT-*Hs*DNPH1 *k*_3_, as predicted based on its role as the general base in the second half-reaction only [6]. In accordance with the conclusions from multiple-turnover experiments (Figure 4C), single-turnover kinetics are compatible with the second half-reaction limiting E55Q-*Hs*DNPH1 *k*_cat_. H56A-*Hs*DNPH1 *k*_3_ is reduced ~12 fold as compared with WT-*Hs*DNPH1 *k*_3_, close to the ~13-fold decrease in *k*_cat_ caused by the H56A substitution. This confirms a predicted role for H56 in the first half-reaction [6], possibly by influencing the p*K*_a_ of groups involved in catalysis [7].

### WT**-** *Hs*DNPH1 reaction rate-limiting step shifts at high pH

**Figure 6:**
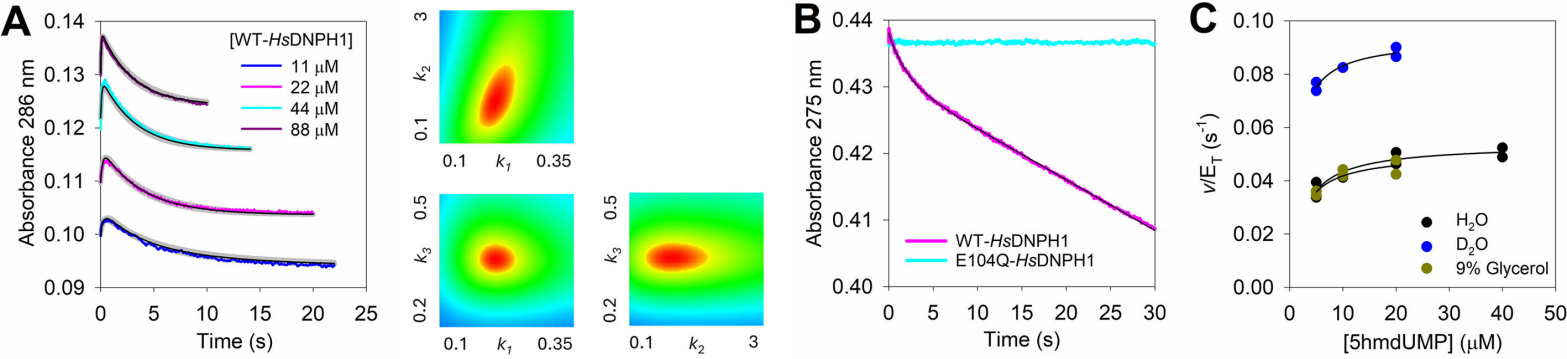
WT-*Hs*DNPH1 kinetics at pH 8.5. (**A**) Single-turnover reaction of WT-*Hs*DNPH1 with 5hmdUMP and FitSpace contour plot of the best-fit model relative to the data. Lines in colour are experimental data, black lines are numerical integration-based fit to a two-step reaction model, and grey lines are boundaries produced by FitSpace analysis. In the contour plots, constrained boundaries are defined by regions in red; *k*_1_ is in units of μM^-1^ s^-1^, and *k*_2_ and *k*_3_, in s^-1^. (**B**) Rapid kinetics under multiple-turnover conditions. Coloured lines are data, and black line is best fit of the data to equation 2. (**C**) Substrate saturation curves in the presence and absence of either glycerol or D_2_O. All data points are shown for 2 independent measurements. lines are best fit of the data to equation 1.

At pH 8.5, the single-turnover kinetics of WT-*Hs*DNPH1 was hypothesised to be slowed down relative to pH 7.0 since the pH-rate profile showed *k*_cat_ is reduced upon deprotonation of a residue with p*K*_a_ of 7.8, likely D80 [7]. Surprisingly, the single-turnover reaction monitored at 286 nm and best fitted to the model in Supplementary Figure S9 (Figure 6A) produced a *k*_3_ slightly higher at pH 8.5 than at pH 7.0, along with a reduced *k*_1_ and a modestly decreased *k*_2_ (Supplementary Table S2). Formation of the E104Q-*Hs*DNPH1-5hmdUMP complex at pH 8.5 led to a differential UV spectrum resembling that at pH 7.0 (Supplementary Figure S10). This is inconsistent with *N*-ribosidic bond cleavage limiting *k*_cat_ at high pH, suggesting a shift in rate-limiting step upon deprotonation of the group with p*K*_a_ of 7.8.

If a step after the first half-reaction chemistry limits *k*_cat_ at pH 8.5, a burst of substrate consumption is predicted under multiple-turnover conditions, and this is in fact observed at 275 nm (Figure 6B). Best fit of the data to equation 2 yielded a *k*_burst_ of 0.494 ± 0.002 s^-1^ and an apparent steady-state rate constant of 0.0452 ± 0.0001 s^-1^. The burst phase amplitude of ~4 μM was just under half the concentration of WT-*Hs*DNPH1 used (10 μM), suggesting half-of-the-sites reactivity is operational for WT-*Hs*DNPH1 catalysis at high pH.

As the pH-rate profile had been obtained employing a composite buffer system, the steady-state kinetics was determined here with the single buffer (Figure 6C) used for pre-steady-state kinetics (100 mM TAPS pH 8.5), showing negligible effects of the buffers used. The *k*_cat_ of 0.054 s^-1^ is in agreement with the apparent steady-state rate constant from rapid kinetics, and the data overlap in the presence and absence of 9% glycerol, indicating diffusional release of either 5hmUra in the first half-reaction or 2-deoxyribose 5-phosphate in the second half-reaction does not limit *k*_cat_ [20]. Moreover, the *k*_cat_ in the presence of D_2_O produced an inverse solvent deuterium isotope effect (^D2O^ *k*_cat_) of 0.58 ± 0.03, in line with what was reported in the pL-rate profiles in H_2_O and D_2_O [7], a likely result of the upward displacement of p*K*_a_s of monoprotic acids in D_2_O from their values in H_2_O [21]. This rules out chemistry in the second half-reaction as the rate-limiting step at pH 8.5 because water activation upon proton abstraction by E55 [6] is expected to occur in the same transition state as the nucleophilic attack on the 5-phospho-2-deoxyribosylated enzyme intermediate, which would result in a normal ^D2O^ *k*_cat_.

### Energy landscape of the *Hs*DNPH1 reaction coordinate

**Figure 7:**
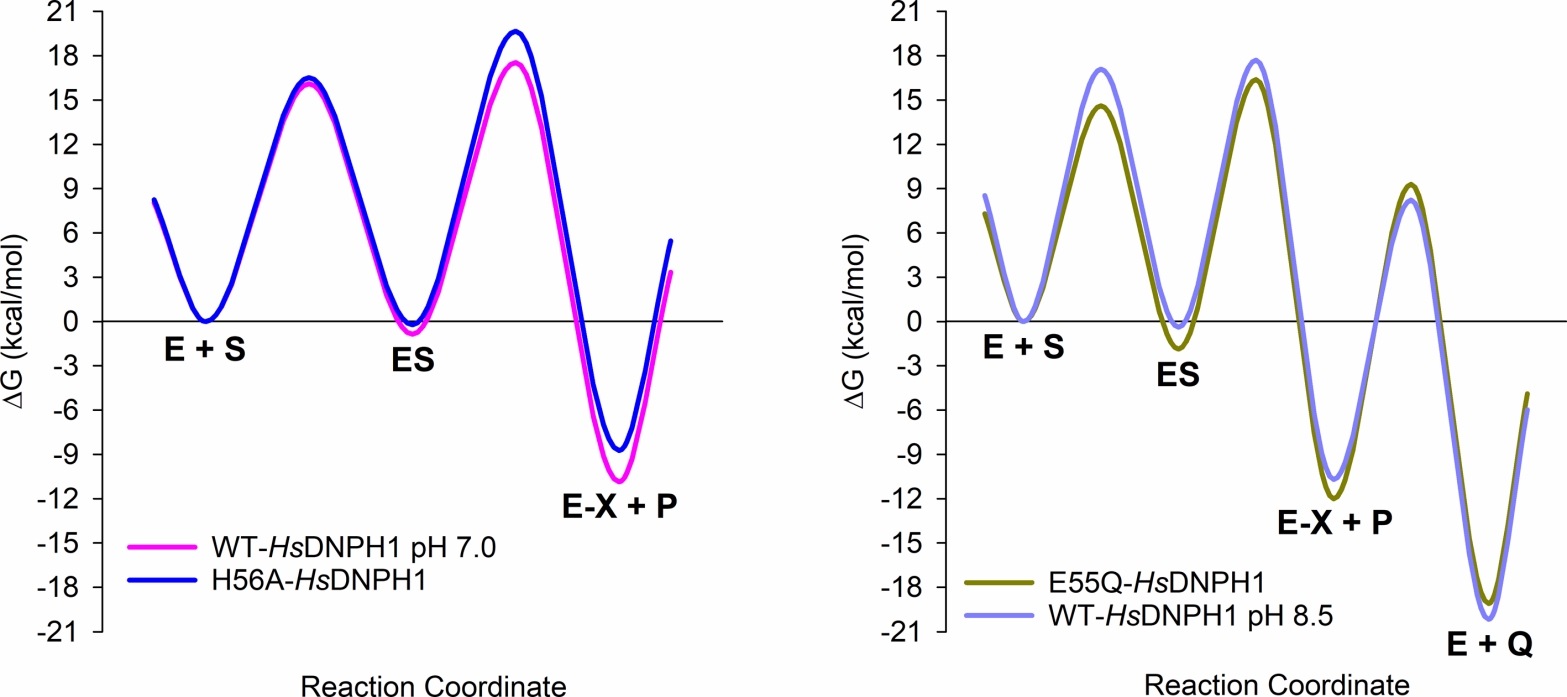
Relative free energy profile of the reaction catalysed by *Hs*DNPH1 variants. E represents enzyme (*Hs*DNPH1 variant), S, substrate (5hmdUMP), ES is the Michaelis complex (*Hs*DNPH1-5hmdUMP), E-X is the covalent intermediate (5-phospho-2-deoxyribosylated enzyme), P is the first product formed (5hmUra), and Q is the last (2-deoxyribose 5-phosphate)

Using the rate constants obtained from single-turnover experiments (Supplementary Table S2), the energy profile along the reaction coordinate for the first half-reaction catalysed by WT- and H56A-*Hs*DNPH1 at pH 7.0 (Figure 7) can be calculated in KinTek based on transition-state theory assuming a recrossing coefficient of unity [18]. The H56A substitution imparts negligible effect on substrate binding, and only a 1.5 kcal/mol increase in the activation energy (ΔG^‡^) relative to WT-*Hs*DNPH1 when moving from the Michaelis complex (ES) to the covalent intermediate and 5hmUra (E-X+P), in excellent agreement with the 1.6 kcal/mol catalytic penalty previously reported from the corresponding *k*_cat_ values [7]. This offers a visual and energy perspective to the conclusion that the first half-reaction defines the kinetics of the overall reaction catalysed by WT-*Hs*DNPH1. It also shows that H56 plays a modest but detectable role in the first half-reaction, as previously suggested [6].

Although the source of the single-turnover signal coupled with the lack of a burst phase precludes the kinetics of the second half-reaction to be gleaned, it is possible to infer the 5-phospho-2-deoxyribosylated enzyme intermediate is fleeting in nature and does not accumulate over the course of the reaction based on the indistinguishable values of *k*_3_ and *k*_cat_. This is in line with the lack of ^D2O^ *k*_cat_ [7], indicating hydrolysis of the covalent intermediate is fast, and also with the lack of solvent viscosity effects [7], suggesting diffusional departure of the products does not limit *k*_cat_. In fact, the products seem to have low affinity for the enzyme forms to which they bind when compared with the substrate affinity. This is concluded from inhibition studies with ribose 5-phosphate as a mimic of 2-deoxyribose 5-phosphate, showing the former is a competitive inhibitor of WT-*Hs*DNPH1 with a *K*_i_ of 6.9 ± 0.8 mM (Supplementary Figure S11A) upon best fit of the data to equation 4, and with 5hmUra, which revealed the IC_50_ to be higher than 400 μM (Supplementary Figure S11B). Interestingly, phosphate is a weak inhibitor of the enzyme with an IC_50_ of 252 ± 16 mM (Supplementary Figure S11C) upon best fit of the data to equation 5.

For E55Q-*Hs*DNPH1 at pH 7.0 and WT-*Hs*DNPH1 at pH 8.5, the shift in rate-limiting step that resulted in the appearance of a burst phase allowed the definition of an additional step in the reaction mechanism upon simultaneous fit of the pre-steady-state data under single- and multiple-turnover conditions to the model in Figure 1B, producing well-constrained FitSpace maps (Supplementary Figure S12) and the rate constants shown in Supplementary Table S3. In Figure 1B, *k*_5_ is a macroscopic rate constant encompassing at least two elementary steps: hydrolysis of the covalent intermediate and release of 2-deoxyribose 5-phosphate from the enzyme. For E55Q-*Hs*DNPH1 at pH 7.0 and WT-*Hs*DNPH1 at pH 8.5, the ΔG^‡^ to move from the ES to the E-X+P complex is ~18.3 kcal/mol and ~18.1 kcal/mol, respectively, very similar to that for WT-*Hs*DNPH1 at pH 7.0. The rate-limiting step is the second half-reaction to regenerate the free enzyme with ΔG^‡^ of ~21.3 kcal/mol for E55Q-*Hs*DNPH1. However, it has contributions from both half-reactions in the case of WT-*Hs*DNPH1 at pH 8.5, since the ΔG^‡^ for the second half-reaction is ~19.0 kcal/mol. The *k*_cat_ in Figure 1B is defined by equation 9, and it can be calculated from the values in Supplementary Table S3. For E55Q-*Hs*DNPH1, this yields a value of 0.00158 s^-1^, an excellent match to the *k*_cat_ of 0.0015 s^-1^ from steady-state data (Supplementary Table S2). For WT-*Hs*DNPH1 at pH 8.5, the calculated value is 0.071 s^-1^, in reasonable agreement with the *k*_cat_ of 0.054 s^-1^ from steady-state data (Supplementary Table S2).


(Equation 9) $$k_{cat}=\frac{k_{3}k_{5}}{k_{3}+k_{5}}$$

### Negative charge develops in the leaving group at the transition state

Formation of the putative anionic 5hmUra intermediate is expected to happen in the step governed by *k*_3_, not in the bimolecular step leading to ES, and is not the source of UV absorbance changes accompanying ES formation. Justification for this conclusion comes from the ^D2O^ *k*_cat_ being unity [7]. If the anionic 5hmUra were produced in an unlikely bimolecular step governed by *k*_1_, its protonation to the neutral 5hmUra would lead to loss and gain of absorbance at 286 nm and 251 nm, respectively, in the slowest step of the reaction. This would yield a sizable normal ^D2O^ *k*_cat_. The simplest explanation is that the anionic 5hmUra is protonated immediately after its formation, making this intermediate too short-lived to produce a distinct kinetic transient.

**Figure 8:**
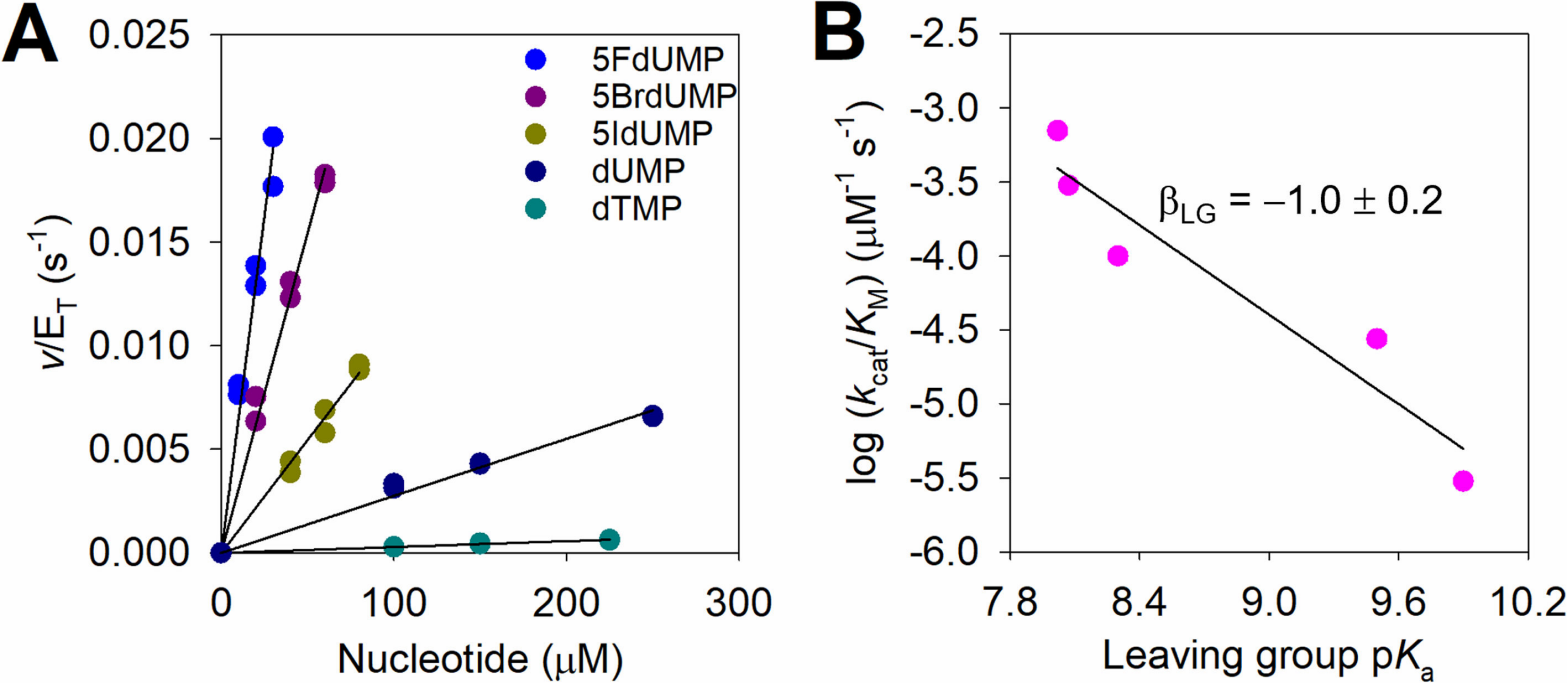
Linear free energy relationships. (**A**) WT-*Hs*DNPH1 reaction rate-dependence on the concentration of different nucleotides. All data points are shown for two independent measurements. Lines are best fit of the data to equation 6. (**B**) Brønsted plots relating *k*_cat_/*K*_M_ with leaving group p*K*_a_. Data are best-fit values ± fitting errors from the analysis in (**A**). Line is best fit of the data to equation 7.

Another explanation is the leaving group in the first half-reaction having neutral charge at the transition state. To test this hypothesis, linear free energy relationships between *k*_cat_/*K*_M_ and leaving group p*K*_a_ were assessed. If *N*-ribosidic bond breaking is rate-limiting for *k*_cat_/*K*_M_ in the first half-reaction and negative charge accumulates in the leaving group moiety at the transition state in comparison with the ground state (free substrate in solution), lowering the leaving group p*K*_a_ should help stabilise the developing negative charge at the transition state, increasing the reaction rate [22,23]. Accordingly, *k*_cat_/*K*_M_ were determined for WT-*Hs*DNPH1 with 5FdUMP, 5BrdUMP, 5IdUMP, dUMP and TMP (in order of increasing p*K*_a_ of the corresponding nucleobase) (Figure 8A, Supplementary Figure S13), upon best fit of the substrate concentration-dependence of the rate to equation 6. The UV absorbance properties of these nucleotides and their corresponding nucleobases, when not available in the literature [15], were determined to inform on the most suitable wavelengths to monitor the reaction and to measure the relevant extinction coefficients (Supplementary Figures S14 and S15); the pH-dependence of the UV absorbance was used to measure the p*K*_a_ of 5-Br-uracil and 5-I-uracil (Supplementary Figure S16).

Analysis of the Brønsted plot (Figure 8B) revealed a strong correlation between decreasing leaving group p*K*_a_ and increasing WT-*Hs*DNPH1 *k*_cat_/*K*_M_, and best fit of the data to equation 7 resulted in a Brønsted slope (β_LG_) of −1.0. β_LG_ of this magnitude suggests significant negative charge development in the leaving group at the transition state [24] and is consistent with a loose transition state in relation to leaving group departure [23]. In the case of the *Hs*DNPH1-catalysed reaction, this is consistent with a transition state for the first half-reaction with advanced C1′–N1 bond cleavage and accumulation of negative charge at the departing 5hmUra.

### Atomistic insight into catalysis by *Hs*DNPH1

**Figure 9:**
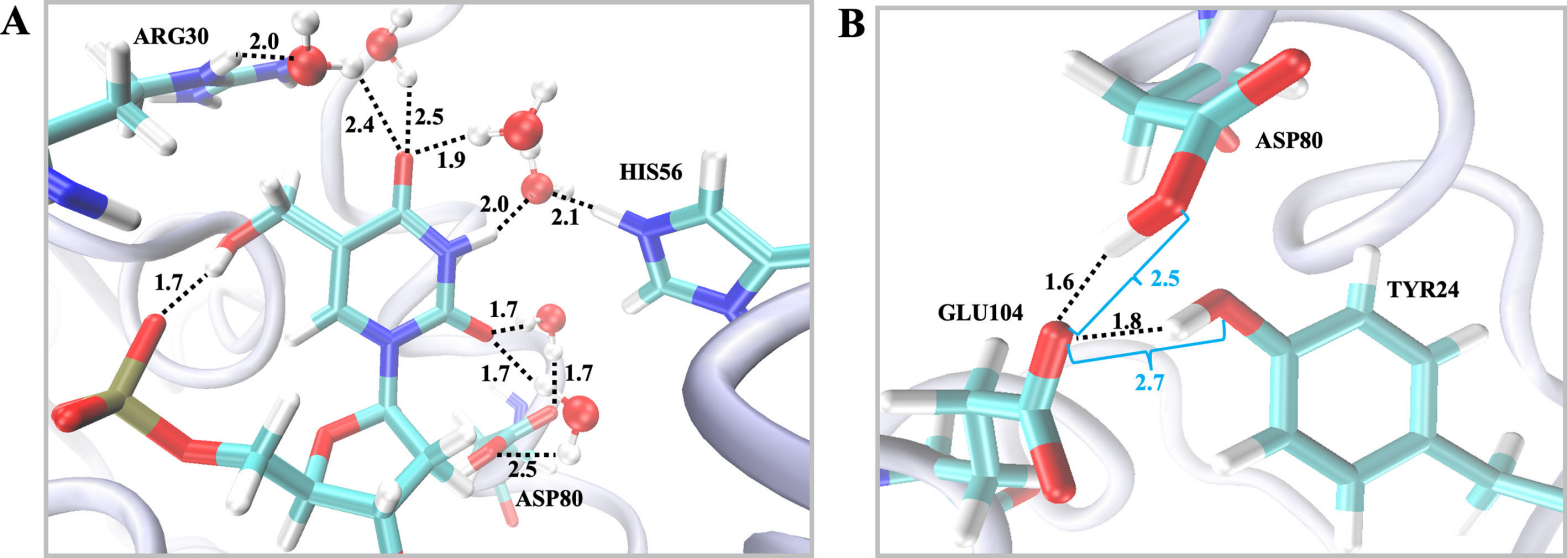
Structure of the solvated *Hs*DNPH1-5hmdUMP complex. (**A**) H-bonding network involving 5hmdUMP’s 5hmUra moiety, water molecules, and key active-site residues. (**B**) Short, strong H-bonds involving the Y24-D80-E104 catalytic triad. Substrate and residue side-chains are represented as sticks, and water molecules as spheres, with atom labels colour-coded with C in cyan, N in blue, O in red, P in yellow, and H in white. The protein backbone is shown as ribbons. Dashed lines are distances in Å.

To provide an atomistic and dynamic model of catalysis for the first half-reaction of *Hs*DNPH1, MD simulations were performed for 8 ns on the equilibrated 5hmdUMP-bound *Hs*DNPH1 structure (PDB ID 8QHQ) [6] (after *in-silico* Q-to-E replacement at position 104 to generate the WT enzyme) simulated in a truncated octahedral box of TIP3P water molecules extending to 12 Å from the protein. The root mean square deviation (RMSD) of the 5hmdUMP-bound *Hs*DNPH1 complex exhibited a rapid increase during the initial phase of the simulation, corresponding to system equilibration. After the initial adjustment period, the RMSD values fluctuated within a narrow range (2.0 Å – 2.5 Å) (Supplementary Figure S17), suggesting minimal conformational change and overall stability of the protein–ligand binding interface throughout the remaining simulation period. The absence of large RMSD deviations of the 8-ns simulation suggests that no major conformational rearrangements occurred, and the complex retained its structural integrity under the simulated conditions. Several enzyme-substrate interactions present in the static *Hs*DNPH1 crystal structures persist in the MD simulation; nevertheless, the latter reveals a highly solvated ground-state structure with interconnected H-bonds involving water molecules that bridge interactions between the 5hmUra moiety and active-site residues spanning D80, H56 and R30 (Figure 9A). We propose that this H-bond network participates in a proton relay that facilitates the rapid protonation of the anionic leaving group following *N*-ribosidic bond cleavage. This H-bond network also offers a mechanistic explanation for the link between the D80A- and H56A-*Hs*DNPH1 pH-rate profiles, which are both flat in contrast to the bell-shaped profile observed with WT-*Hs*DNPH1 [7]. Interestingly, the short, strong H-bonds among residues of the catalytic triad – Y24-D80-E104 – seen in crystal structures [5,7] are maintained during the simulations (Figure 9B; Supplementary Figure S18). These H-bonds are proposed to modulate the nucleophilicity of E104 and position it for nucleophilic attack [7]. This hypothesis is supported by the observed donor-acceptor distances of 2.7 Å between Y24 and E104, and 2.5 Å between D80 and E104, which aid in positioning the E104 carboxylate group ~3.6 Å from the C1′ of 5hmdUMP.

**Figure 10:**
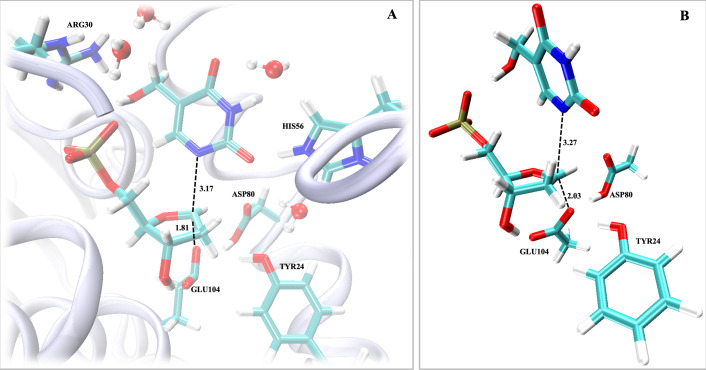
Transition-state structures for 5hmdUMP *N*-ribosidic bond cleavage. (**A**) QM/MM-calculated ((ONIOM (B3LYP-D3(BJ)/6–31+g*/UFF) on-enzyme transition-state structure of the *Hs*DNPH1 first half-reaction. Atoms in stick and in sphere representations were included in the QM region. (**B**) DFT-calculated (B3LYP-D3(BJ)/6–31+g* CPCM solvent = water) off-enzyme transition-state model of the *Hs*DNPH1 first half-reaction. Substrate and residue side chains (or side-chain mimics) are represented as sticks, and water molecules as spheres, with atom labels color-coded with C in cyan, N in blue, O in red, P in yellow, and H in white. The protein backbone is shown as ribbons. Dashed lines are distances in Å.

The chemistry of the first half-reaction was calculated utilising QM/MM ONIOM (B3LYP-D3(BJ)/6–31+g*/UFF) for the on-enzyme transition-state structure starting from the explicitly solvated MD equilibrated geometry, and using DFT (B3LYP-D3(BJ)/6–31+g* CPCM solvent = water) calculations to locate the off-enzyme transition-state structure with a model that includes mimics of the Y24, D80 and E104 side-chains. The lowest energy calculations for both the on- and off-enzyme transition-state structures are consistent with a concerted, S_N_2-like transition state (Figure 10), with advanced C1′−N1 bond-breaking distances (r_C−N_) and C1−E104 carboxylate O bond-forming distances (r_C−O_). A stepwise, S_N_1-like transition state could not be located on either surface. The on-enzyme transition-state structure is characterised by both advanced anionic 5hmUra departure (r_C−N_ of 3.17 Å) and nucleophilic attack (r_C−O_ of 1.81 Å) (Figure 10A), which differs slightly from the off-enzyme transition-state structure with a more elongated leaving group distance (r_C−N_ of 3.27 Å) but less advanced nucleophilic attack (r_C−O_ of 2.03 Å) (Figure 10B). The advanced bond-breaking and bond-forming distances observed for the lowest energy on-enzyme transition-state structure are consistent with a highly stabilised transition state due to active site interactions and are consistent with the conclusions from the Brønsted plot analysis.

The calculated free energy barrier for the on-enzyme reaction (ΔG^‡^ between the Michaelis complex and the transition state for the first half-reaction) is ~21.9 kcal/mol, which is in reasonable agreement with the experimental single-turnover rate constant-derived ΔG^‡^ (which according to our proposed kinetic model reflects the same complexes) of ~18.4 kcal/mol, although caution should be wielded when interpreting absolute energy barriers derived from ONIOM calculations [25]. As expected, the enzyme provides substantial stabilisation of the transition state, consistent with the increased off-enzyme reaction ΔG^‡^ of ~33.2 kcal/mol. This increase in ΔG^‡^ likely stems from the exclusion of R30 and H56 from the off-enzyme transition state.

**Figure 11:**
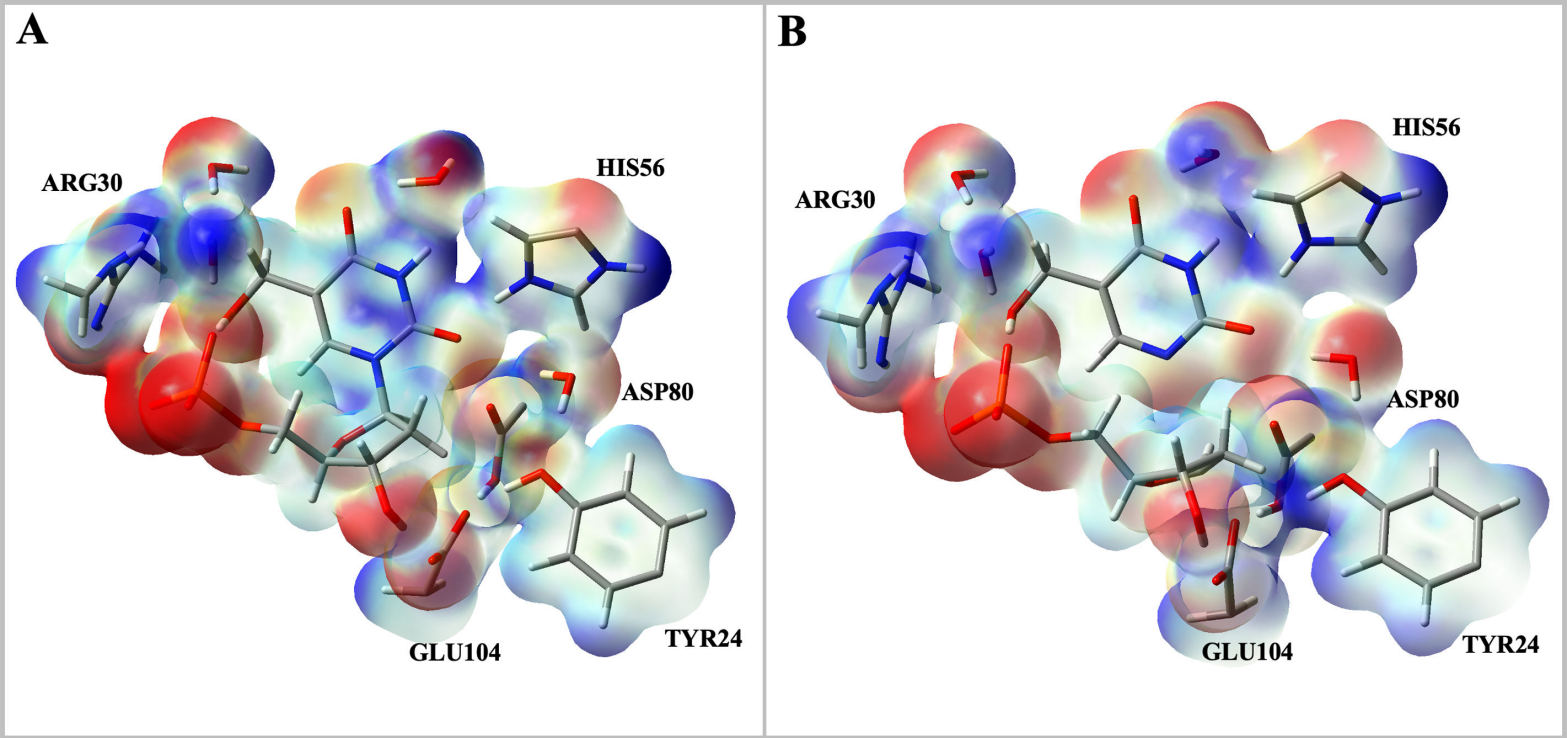
Molecular electrostatic potential maps. (**A**) Michaelis complex molecular electrostatic potential map. (**B**) Transition-state complex molecular electrostatic potential map. The charge gradient varies from positive charge (blue surface) to negative charge (red surface).

Molecular electrostatic potential maps were calculated for both the Michaelis complex and the on-enzyme transition state complex (Figure 11). The molecular electrostatic potential map of the Michaelis complex shows accumulation of positive charge (blue surface) on the 5hmUra moiety of 5hmdUMP, on R30, and, to a lesser extent, on H56, whereas negative charge (red surface) is concentrated on the nucleophilic E104, and on the PO_4_^2−^ moiety of 5hmdUMP (Figure 11A). In contrast, the transition-state complex reflects a reduction of the negative charge on E104 and accumulation of negative charge on the departing 5hmUra (Figure 11B). The surrounding positively charged R30 and H56 likely stabilise the negative charge developing on the leaving group at the transition state. It should be pointed out that even though the molecular electrostatic potential maps were calculated solely from the QM region and therefore do not include possible polarisation effects from the MM region, both maps (for ground state and transition state) were calculated the same way, allowing a comparison between them.

## Discussion

*Hs*DNPH1 catalysis proceeds via a double-displacement mechanism involving two half-reactions [5,6], but which of these half-reactions limits the overall reaction rate, an important step to uncover kinetic bottlenecks along the reaction coordinate, was not resolved. This work answered this question, enabled initially by the discovery that 5hmdUMP binding to *Hs*DNPH1 causes a spectral shift on the Michaelis complex before chemistry occurs, which allowed a clear distinction of spectral signals arising from binding and *N*-ribosidic bond cleavage. Multiple-turnover rapid kinetics with WT-*Hs*DNPH1 and the catalytically inactive E104Q-*Hs*DNPH1 revealed that the apparent burst phase of the reaction at 264 nm is due wholly to substrate binding, not C1′−N1 bond cleavage. This scenario is reminiscent of the apparent burst phase of SARS-CoV-2 3-chymotrypsin-like protease catalysis with a fluorescently labelled peptide substrate, which was shown with a catalytically inactive variant to be caused by fluorescence change upon binding before covalent attack by the catalytic cysteine residue [26]. Monitoring the reaction at a wavelength (275 nm) where binding does not lead to 5hmdUMP absorbance change made it clear that *Hs*DNPH1 catalysis does not involve burst kinetics, suggesting no rate-limiting step takes place after 5hmUra formation.

Hypochromism upon nucleotide binding to proteins often results from π-stacking interactions between the nucleobase and aromatic groups in the protein, while red shift of the λ_max_ has been attributed to the formation of H-bonds between protein and nucleobase [27]. A red-shift of ~20 nm of the flavin-adenine dinucleotide spectrum was attributed to an H-bond between the cryptochrome 4 protein and the cofactor [28]. From *Hs*DNPH1-5hmdUMP complex crystal structures, H-bonds involving the 5hmUra moiety and N80 (in the D80N-*Hs*DNPH1 variant) [7] or H56 (in the E104Q-*Hs*DNPH1 variant) [6], and a water bridge between the 5hmUra moiety and R30 [6,7], can be inferred, but no π-stacking interaction is apparent from the crystal structures [6,7] or the MD simulations. The dielectric constant of the medium may also affect the absorbance properties of molecules [29], and enzyme active sites often have lower overall dielectric constants than bulk water [30]. Therefore, a combination of specific H-bonds and a lower dielectric constant environment upon enzyme binding may contribute to the differential spectra of enzyme-bound 5hmdUMP.

The ITC-based equilibrium binding data and the magnitude of the burst phase at pH 8.5 raised the possibility of half-of-the-sites binding/catalysis, a relatively common phenomenon in *N*-ribosidic bond-breaking enzymes. For instance, *E. coli* and *Staphylococcus aureus* dimeric 5′-methylthioadenosine *N*-hydrolases are inverting *N*-hydrolases which show half-of-the-sites reactivity and transition-state analogue binding [31,32]. Nevertheless, this question is not yet settled with *Hs*DNPH1 due to the lack of burst kinetics with the WT enzyme at optimal pH (7.0). If confirmed by further experiments (e.g. inhibition titration with a tight-binding substrate analogue), half-of-the-sites catalysis may have implications for inhibitor development, as inhibiting only half of the active sites might be sufficient to abolish catalysis altogether.

Global analysis of binding and single-turnover kinetics at different wavelengths established the kinetics of the first half-reaction, encompassing one-step substrate binding and one-step 5hmUra formation, as sufficient to account for the kinetics of the entire catalytic cycle. The proposed anionic 5hmUra intermediate [5–7] did not produce a distinct kinetic transient, probably being too short-lived and rapidly protonated to the neutral product 5hmUra. An analogous scenario was proposed for the reaction catalysed by tryptophan synthase, in which out of three chemically logical intermediates, only one was long-lived enough to be kinetically significant, and the four-step reaction could be described by a bimolecular binding step and a unimolecular chemical step [18,33].

In *lieu* of kinetic evidence for an anionic 5hmUra intermediate, evidence for significant anionic character of the leaving group and extensive C1′−N1 bond breaking at the transition state was gleaned from the large and negative β_LG_. This was corroborated by QM/MM calculations, which readily located a transition state quantifying these features, besides pointing to strong participation of the nucleophilic E104 in an S_N_2-like transition state. The positions of the anionic 5hmUra and 5-phospho-2-deoxyribosyl moieties at the ground and transition states lend support to the recently proposed mechanism of nucleophilic substitution by electrophile migration, based on crystal structures with transition-state analogues [8]. A combination of kinetic isotope effects and computational chemistry has also indicated an anionic uracil leaving group at the transition states of the reactions catalysed by uridine phosphorylase [34] and uracil DNA glycosidase [35]. Further support for the proposed transition state for the *Hs*DNPH1-catalysed reaction could emerge from kinetic isotope effects measurement, particularly to quantify the extent of nucleophile (E104) participation. Such information, along with the details provided here on the nature of the rate-limiting step, could serve as a blueprint to aid inhibitor design.

## Methods

### Materials

All commercially available materials were purchased from Merck, Roche, Expedeon, Formedium, Agilent Technologies, Abcam, Cytiva, Jena Bioscience and ThermoFisher Scientific, and used as received unless otherwise stated. *Escherichia coli* 5-alpha and BL21(DE3) competent cell lines used for plasmid preparation and protein expression respectively were from New England Biolabs. DNA oligonucleotide primers were from Integrated DNA Technologies (IDT). Production of WT-, H56A- and E104A-*Hs*DNPH1 variants, and synthesis of 5-hydroxymethyl-2′-deoxyuridine 5′-phosphate were carried out as previously described [7].

### Site-directed mutagenesis of *Hs*DNPH1

Site-directed mutagenesis was carried out with overlapping primers according to the method of Liu and Naismith [36]. Oligonucleotide primer sequences are listed in Supplementary Table S4. The following single-, double- and triple-amino-acid substitutions were produced: E104Q-, E55Q-, H56A/E104Q-, R30A/E104Q-, H56A/D80A/E104Q-*Hs*DNPH1. WT-*Hs*DNPH1 expression plasmid [5] was used as the DNA template for single-amino-acid mutations, E104Q-*Hs*DNPH1 expression plasmid was used as the template for double-amino-acid mutations, and H56A/E104Q-*Hs*DNPH1 expression plasmid for the triple-amino-acid mutation. Correct insertion of each mutation was confirmed by DNA sequencing (Eurofins Genomics). All *Hs*DNPH1 variants were produced as previously described [7].

### UV-VIS absorbance spectra of *Hs*DNPH1-catalysed reaction

All spectra were acquired independently at 25°C in a 1 cm path length quartz cuvette (Hellma) using a Shimadzu UV-2600 spectrophotometer outfitted with a CPS unit for temperature control. Measurements were taken every 1 nm from 220 to 400 nm, and three independent measurements were averaged. Reactions (500 μl) for conversion of 5hmdUMP to 5hmUra and 2-deoxyribose 5-phosphate by *Hs*DNPH1 contained 100 mM HEPES pH 7.0, 20 μM 5hmdUMP, and 0.4 μM either WT- or H56A-*Hs*DNPH1. Spectra were acquired 10 s after enzyme addition and either every 30 s (WT-*Hs*DNPH1) or 9 min (H56A-) thereafter until the reaction reached completion. A baseline of all reaction components bar enzyme was taken prior to reaction. Spectra of 20 μM either 5hmdUMP or 5hmUra in 100 mM HEPES pH 7.0 and 50 mM NaH_2_PO_4_ pH 12.0 were also acquired, baselined with the corresponding buffer prior to data acquisition.

### UV-VIS absorbance spectra of 5-substituted nucleotides and nucleobases

UV-VIS absorbance spectra of 1.34 mM 5 F-2′-deoxyuridine 5′-phosphate (5FdUMP) and 5-F-uracil, 2.6 mM 5-Br-2′-deoxyuridine 5′-phosphate (5BrdUMP) and 5-Br-uracil, 2.1 mM 5-I-2′-deoxyuridine 5′-phosphate (5IdUMP) and 5-iodo uracil, and 1.0 mM 2′-deoxythymidine 5′-phosphate (TMP) and thymine were measured independently in duplicate in a NanoDrop spectrophotometer every 0.5 nm from 190 to 850 nm and averaged. Nucleotides were suspended in H_2_O and nucleobases in DMSO, and a baseline measurement with the corresponding solvent was taken prior to each spectrum measurement. Nucleobase spectra were subtracted from the corresponding nucleotide spectra, and wavelengths with the greatest ΔAbs were identified for use in kinetic studies.

### Determination of extinction coefficients

All absorbance measurements were carried out in a NanoDrop spectrophotometer in three independent measurements. The absorbance at 251, 275 and 286 nm of 5hmdUMP in 100 mM HEPES pH 7 and 5hmUra in 100 mM HEPES pH 7 and 50 mM NaH_2_PO_4_ pH 12 were determined at known concentrations, and standard curves were used to calculate extinction coefficients. Known concentrations of each analyte were determined spectrophotometrically at 264 nm for 5hmdUMP (ɛ_264_ = 10,200 M^-1^ cm^-1^) [15] in 100 mM HEPES pH 7 (5hmdUMP concentrations ranged from 0.060 to 0.46 mM for experiments at 251 and 286 nm and 0.12–0.86 mM for the experiment at 275 nm), 261 nm for 5hmUra in 100 mM HEPES pH 7 (ɛ_261_ = 8100 M^-1^ cm^-1^) [15] (concentrations ranged from 0.039 to 0.73 mM for experiments at 251 and 286 nm and 0.059–0.73 mM for the experiment at 275 nm), and 286 nm for 5hmUra in 50 mM NaH_2_PO_4_ pH 12 (ɛ_286_ = 7400 M^-1^ cm^-1^) [15] (concentrations ranged from 0.050 to 0.72 mM for the experiment at 251 nm and 0.039–0.74 mM for the experiment at 275 nm). Absorbances were measured at 273 nm for 5FdUMP and 5-F-uracil at known concentrations (0.28–1.6 mM 5FdUMP determined spectrophotometrically at 267 nm, ɛ_267_ = 10,000 M^-1^ cm^-1^, Jena Bioscience; and 0.2–1.6 mM 5-F-uracil determined by mass), at 290 nm for 5BrdUMP and 5-Br-uracil (0.30–2.3 mM 5BrdUMP determined spectrophotometrically at 278 nm, ɛ_278_ = 9700 M^-1^ cm^-1^, Jena Bioscience; and 0.2–1.6 mM 5-Br-uracil determined by mass), at 303 nm for 5IdUMP and 5-I-uracil (0.17–1.4 mM 5IdUMP determined spectrophotometrically at 303 nm, ɛ_303_ = 7700 M^-1^ cm^-1^, Jena Bioscience; and 0.2–3.2 mM 5-I-uracil determined by mass), and at 276 nm for TMP and thymine (0.27–2.2 mM TMP determined spectrophotometrically at 267 nm, ɛ_267_ = 9600 M^-1^ cm^-1^ and 0.26–2.1 mM thymine determined spectrophotometrically at 264 nm, ɛ_264_ = 7900 M^-1^ cm^-1^) [15]. Standard curves were used to calculate the extinction coefficients at the respective wavelengths.

### Spectrophotometric assay of *Hs*DNPH1 activity under steady-state conditions

Initial rates of enzyme-catalysed hydrolytic reactions (500 μl) of nucleotides at 25°C in 100 mM HEPES pH 7.0 were monitored continuously in 1 cm optical path length quartz cuvettes (Hellma) in a Shimadzu UV-2600 spectrophotometer outfitted with a CPS unit for temperature control. Concentrations of 5hmdUMP and enzyme were 1.125–40 μM 5hmdUMP and either 0.1 μM WT-*Hs*DNPH1 (1.25 μM 5hmdUMP), 0.2 μM WT-*Hs*DNPH1 (2.5–5 μM 5hmdUMP) or 0.4 μM WT-*Hs*DNPH1 (10–40 μM 5hmdUMP); 5–40 μM 5hmdUMP and either 1 μM H56A-*Hs*DNPH1 (5 μM 5hmdUMP) or 2 μM H56A-*Hs*DNPH1 (10–40 μM 5hmdUMP); 20–60 μM 5hmdUMP and 15 μM E55Q-*Hs*DNPH1. Activity of WT-*Hs*DNPH1 was assayed for 120 s, activity of E55Q-*Hs*DNPH1, for 600 s, and activity of H56A-*Hs*DNPH1, for up to 180 s. Concentrations of alternative nucleotides and enzyme were 10–30 μM 5FdUMP and 1 μM WT-*Hs*DNPH1; 20–60 μM 5BrdUMP and 2 μM WT-*Hs*DNPH1; 40–80 μM 5IdUMP and 3 μM WT-*Hs*DNPH1; 100–250 μM dUMP and 20 μM WT-*Hs*DNPH1; 100–225 μM TMP and 20 μM WT-*Hs*DNPH1. Activity was assayed for up to 600 s. Alternatively, initial rates of WT-*Hs*DNPH1-catalysed hydrolysis of 5hmdUMP were measured for 3 min in 100 mM TAPS pH 8.5 (0.6 μM WT-*Hs*DNPH1 and 5 μM 5hmdUMP; 0.8 μM WT-*Hs*DNPH1 and 10–40 μM 5hmdUMP) and under the same conditions in the presence of 9% glycerol. Initial rates in 98.8% D_2_O were measured in 100 mM TAPS pD 8.5 (0.3 μM WT-*Hs*DNPH1 and 5 μM 5hmdUMP; 0.6 μM WT-*Hs*DNPH1 and 10 μM 5hmdUMP; 0.8 μM WT-*Hs*DNPH1 and 20 μM 5hmdUMP). Cuvettes were incubated in the spectrophotometer at 25°C for 3 min before the reaction was initiated by the addition of enzyme.

For inhibition studies in 100 mM HEPES pH 7.0, initial rates of enzyme-catalysed hydrolysis of either 1.25–50 μM 5hmdUMP in the presence of 0–400 μM 5hmUra, 10 μM 5hmdUMP and 0–400 mM H_2_NaPO_4_ pH 7.0, or 1.25–80 μM 5hmdUMP in the presence of 0–60 mM ribose 5-phosphate were measured with 0.1 μM WT-*Hs*DNPH1 (1.25 μM 5hmdUMP), 0.2 μM WT-*Hs*DNPH1 (2.5–5 μM 5hmdUMP) or 0.4 μM WT-*Hs*DNPH1 (10–40 μM 5hmdUMP) for 120 s. Cuvettes were incubated in the spectrophotometer at 25°C for 3 min before reaction was initiated by the addition of 5hmdUMP. Controls lacked enzyme.

Two independent measurements were performed, except for WT-*Hs*DNPH1 at pH 7.0, where four independent measurements were carried out. Controls lacked enzyme. Absorbance decrease upon nucleotide *N*-ribosidic bond hydrolysis was monitored at 275 nm with 5hmdUMP (Δɛ_275_ = 3539 M^-1^ cm^-1^), 273 nm with 5FdUMP (Δɛ_273_ = 3489 M^-1^ cm^-1^), 290 nm with 5BrdUMP (Δɛ_290_ = 2100 M^-1^ cm^-1^), 303 nm with 5IdUMP (Δɛ_303_ = 2014 M^-1^ cm^-1^), 276 nm with TMP (Δɛ_276_ = 2338 M^-1^ cm^-1^), reported in this work, and 282 nm with dUMP (Δɛ_282_ = 1600 M^-1^ cm^-1^) [37].

### Measurement of p*K*_a_s of 5-Br-uracil and 5-I-uracil

UV-VIS absorbance spectra of 5-Br-uracil and 5-I-uracil (500 μM) in 100 mM MES/HEPES, 50 mM NaH_2_PO_4_ (5% DMSO) were measured independently in triplicate in a Nanodrop spectrophotometer every 0.5 nm from 190 to 850 nm from pH 6.0–11.0 and averaged. A baseline measurement with the corresponding buffer was taken prior to each spectrum measurement. Absorbance at 300 nm for 5-Br-uracil and at 305 nm for 5-I-uracil was plotted against pH as a proxy for anionic nucleobase formation.

### HPLC

A reaction mix (100 μl) containing 100 mM HEPES pH 7.0, 200 μM 5hmdUMP and 10 μM E104Q-*Hs*DNPH1 was incubated at 25°C for 4 h, quenched with 200 μl of ice-cold methanol, vortexed for 2 min and centrifuged (16,000 ***g***) for 10 min. The supernatant was collected, dried under vacuum centrifugation and resolubilised in 100 μl HPLC-grade water. The HPLC analysis was carried out as previously reported [7].

### ITC

ITC measurements were carried out at 25°C in a MicroCal PEAQ-ITC calorimeter (Malvern Instruments). E104Q*-Hs*DNPH1 was buffer exchanged into 100 mM HEPES pH 7.0 using a Vivaspin 20 centrifugal concentrator and 5hmdUMP diluted using the corresponding filtrate to ensure an exact buffer match between enzyme and substrate. After a small injection of 0.4 μl, 18 successive injections of 2 μl 5hmdUMP (250 μM) were made into 300 μl of 30 μM *Hs*DNPH1 with 120 s spacing between injections and a reference power of 10 μcal s^-1^. Two independent experiments were carried out. Heat of dilution was measured by titrating 5hmdUMP into buffer and subtracted from the binding curves. Data were fitted to a single-site binding model with 2:1 (protein:ligand) stoichiometry using the PEAQ-ITC analysis software (Malvern Instruments).

### Differential *Hs*DNPH1-5hmdUMP binding spectra

UV-VIS absorbance spectra of 5hmdUMP in the presence and absence of inactive *Hs*DNPH1 variants were acquired at 25°C in a 1 cm path length, 2-chamber quartz cuvette with an internal mixing window (Hellma) using a Shimadzu UV-2600 spectrophotometer outfitted with a CPS unit for temperature control. One chamber (1 ml) contained 5hmdUMP, while the other, enzyme (40 μM 5hmdUMP, 60 μM E104Q-*Hs*DNPH1; 60 μM 5hmdUMP, 120 μM H56A/E104Q-*Hs*DNPH1; 40 μM 5hmdUMP, 120 μM R30A/E104Q-*Hs*DNPH1; 60 μM 5hmdUMP, 120 μM H56A/D80A/E104Q-*Hs*DNPH1) either in 100 mM HEPES pH 7, or, for the WT-*Hs*DNPH1 experiment only, also in 100 mM TAPS pH 8.5. The UV-VIS spectra of 5hmdUMP in water and in ethanol were measured independently using the same cuvette, and the former was subtracted from the latter to obtain the difference spectrum in ethanol. Absorbance was measured every 1 nm from 220 to 400 nm prior to mixing of enzyme and substrate, and again after the two chambers had been mixed through the internal cuvette window. The chambers were mixed again, and the measurement repeated. The spectrum of free 5hmdUMP and enzyme (the spectrum taken prior to mixing) was subtracted from the spectrum of enzyme-bound 5hmdUMP (the spectrum acquired after mixing).

### General assay for rapid kinetics

Assays for multiple- and single-turnover kinetics and binding kinetics at 25°C were carried out by monitoring the change in absorbance at either 251, 264, 275 or 286 nm upon rapid mixing of 5hmdUMP and enzyme in an Applied Photophysics SX-20 stopped-flow spectrophotometer outfitted with a 5 μl mixing cell (0.5 cm path length, 0.9 ms dead time) and a circulating water bath for temperature control. In every experiment, one syringe contained *Hs*DNPH1 while the other contained 5hmdUMP. *Hs*DNPH1 was buffer exchanged into either 100 mM HEPES pH 7.0 or 100 mM TAPS pH 8.5 using a Vivaspin 20 centrifugal concentrator, and 5hmdUMP was diluted using the corresponding filtrate to ensure an exact buffer match between enzyme and substrate. Reactions were triggered by rapidly mixing 55 μl from each syringe. Control reactions lacked either enzyme or substrate.

### Single-turnover kinetics

The enzyme-catalysed hydrolysis of 10 μM 5hmdUMP under single-turnover conditions was monitored at either 251 nm (11–167 μM WT-*Hs*DNPH1), or 286 nm (11–88 μM WT-*Hs*DNPH1; 11–88 μM H56A-*Hs*DNPH1; 11–88 μM E55Q-*Hs*DNPH1), with 10,000 data points collected over a maximum of either 15 s (WT-*Hs*DNPH1 and E55Q-*Hs*DNPH1) or 180 s (H56A-*Hs*DNPH1) at pH 7.0, and over a maximum of 22 s for WT-*Hs*DNPH1 at pH 8.5, in a logarithmic timescale. Between three and six traces were averaged for each reaction.

### Multiple-turnover kinetics

The enzyme-catalysed hydrolysis of 5hmdUMP under multiple-turnover conditions was monitored at either 275 nm (10 μM WT-*Hs*DNPH1 and 100 μM 5hmdUMP; 10 μM E55Q-*Hs*DNPH1 and 50 μM 5hmdUMP) or 264 nm (10 μM WT-*Hs*DNPH1 and 100 μM 5hmdUMP). For WT-*Hs*DNPH1 at pH 7.0, 3252 data points were collected either for 1.9 s at 275 nm in a logarithmic timescale or for 1.5 s at 264 nm in a split-time basis with 5000 data points acquired in the first 0.1875 s, and 5000 data points acquired in the remaining 1.3125 s. A minimum of four traces were averaged for each reaction. For E55Q-*Hs*DNPH1, 10,000 data points were acquired for 1 min in a logarithmic timescale, and three traces were averaged. For WT-*Hs*DNPH1 at pH 8.5, 10,000 data points were collected for 30 s at 275 nm in a logarithmic timescale, and seven traces were averaged.

### Binding kinetics

In the first set of experiments, the binding kinetics of 10 μM 5hmdUMP to *Hs*DNPH1 catalytically inactive variants were monitored at either 251 nm (2–88 μM E104Q-*Hs*DNPH1) or 286 nm (3–88 μM E104Q-*Hs*DNPH1; 11–88 μM H56A/E140Q-*Hs*DNPH1) with 10,000 data points collected over a maximum of either 15 s in a logarithmic timescale with a minimum of three traces averaged per reaction. In the second set of experiments, the binding kinetics of 100 μM 5hmdUMP to 10 μM E104Q-*Hs*DNPH1 was monitored at either 275 nm for 2 s in a logarithmic timescale with 10,000 data points collected or 264 nm for 1.5 s in a split-time basis with 5000 data points acquired in the first 0.1875 s, and 5000 data points acquired in the remaining 1.3125 s, with a minimum of four traces averaged for each reaction.

### Kinetics data analysis

Analytical data fitting employed the nonlinear regression function of SigmaPlot 13.0 (SPSS). Substrate saturation curves under pseudo-first-order conditions were fitted to equation 1. Multiple-turnover kinetics data with a burst of substrate consumption were fitted to equation 2. Substrate saturation curves not under pseudo-first-order conditions were fitted to equation 3. Inhibition data were fitted to either equation 4 or equation 5. Substrate dependence of the rate at very low substrate concentrations only was fitted to equation 6. Brønsted plots were fitted to equation 7. In equations 1 – 7, *v* is the initial rate, *K*_M_ is the Michaelis constant, *k*_cat_ is the steady-state catalytic constant, *S* is the substrate concentration, *E*_T_ is the total enzyme concentration, *A*(t) is the absorbance at time *t*, *A*(0) is the absorbance at *t* = 0, and *k*_burst_ is the observed rate constant for the burst phase, *b*_LG_ is the Brønsted coefficient, *K*_a_ is the acid dissociation constant, log (C) is the y-axis intercept, *I* is the concentration of inhibitor, *K*_i_ is the inhibition constant, *v*_i_ and *v*_0_ are the initial rate in the presence and absence of inhibitor, respectively, IC_50_ is the half-maximal inhibitory concentration, *h* is the Hill coefficient.

Rapid kinetics data were fitted to reaction models by numerical integration as implemented in KinTek Global Explorer [16,18] and the best fitted values were evaluated for how constrained they were by the data and model via FitSpace analysis [17,18]. After exporting the data, the rate constant depiction (*k*_n_) in the FitSpace axes was changed so that *n* = odd number represents a forward reaction step, and *n* = even number represents a reverse reaction step. A one-step binding model (E + S = ES) was used to fit binding kinetics, a two-step reaction model (E + S = ES = E X + P) was used to fit single-turnover kinetics, and a three-step reaction model (E + S = ES = E X + P = E + Q) was used for global fit of single- and multiple-turnover kinetics when the latter was characterised by a burst phase. In all models, E and S are free enzyme and free substrate, respectively, P and Q are the first and second products formed, respectively, ES and E-X are the Michaelis complex and the 5-phospho-2-deoxyribosylated enzyme intermediate, respectively, and chemistry in the first half-reaction and release of the first product (ES = E X + P) and chemistry in the second half-reaction and release of the second product (EX = E + Q) were treated as irreversible steps. Signal observables were defined at 251 nm by the loss of absorbance upon ES formation and regain of absorbance upon *N*-ribosidic bond cleavage; at 286 nm by the gain of absorbance upon ES formation and loss of absorbance upon *N*-ribosidic bond cleavage; at 275 nm by the loss of absorbance upon *N*-ribosidic bond cleavage.


(Equation 1) $$\frac{v}{E_{T}}=\frac{k_{cat}S}{K_{M}+S}$$

(Equation 2) $$A\left(t\right)=A\left(0\right)e^{-k_{burst}t}+vt$$

(Equation 3) $$\frac{v}{E_{T}}=k_{cat}\frac{E_{T}+S+\;K_{M}-\;\sqrt{{\left(E_{T}+S+K_{M}\right)}^{2}-4E_{T}S}}{2E_{T}}$$

(Equation 4) $$\frac{v}{E_{T}}=\;\frac{k_{cat}S}{\left(1+\;\frac{I}{K_{i}}\right)K_{M}+S}$$

(Equation 5) $$\frac{v_{i}}{v_{0}}=\frac{1}{1+{\left(\frac{I}{IC_{50}}\right)}^{h}}$$

(Equation 6) $$\frac{v}{E_{T}}=\frac{k_{cat}S}{K_{M}}$$

(Equation 7) $$\log\left(\frac{k_{cat}}{K_{M}}\right)=\beta_{LG}pK_{a}+\log\left(C\right)$$

### QM and QM/MM methods

All the calculations in this study have been performed with a full quantum mechanical (QM) density-functional theory (DFT) as well as ONIOM [38] (QM/MM) approach, with the aid of the Gaussian 16 suite of programs [39] using the B3LYP functional [40]. The 6–31+g* [41] basis set has been employed for all the energy minimisations and transition state searches. Solvent corrections were incorporated with optimisation calculations using the integral formalism variant polarisable continuum model (IEFPCM) with water (*ε* = 80) as the solvent. Further, we performed the ONIOM (QM/MM) with the substrate-bound full protein system. The starting point of the ONIOM (QM/MM) calculation was the equilibrated geometry from the molecular dynamics (MD) simulation (details below). The QM region includes the 5hmdUMP substrate and all the key residues involved directly in catalysis of the first half-reaction, Y24, protonated D80, E104, protonated (Nδ1) H56 and R30, along with four water molecules that make a hydrogen bonding network with the substrate and the amino acid residues. This consists of a total of 87 atoms in the QM region (see Figure 10A, the QM region shown with the sticks model). The universal force field (UFF) [42] was employed for the low-layer MM calculation. UFF is a general-purpose force field applicable to a wide range of elements, including proteins and biomolecules. Similar approaches have also been used in our previous calculations in biocatalysis [43]. The quadratic coupled algorithm [44] and the mechanical embedding scheme were used in geometry optimisation, where QM calculations are performed on the QM region without explicitly including the electrostatic potential of the MM region, as our calculation focused mainly on the interactions between the QM and MM regions which are handled purely at the MM level, including bonded and non-bonded interactions like electrostatic and van der Waals forces. The values reported are ΔG values, with zero-point energy corrections, internal energy and entropic contributions included through frequency calculations on the optimised minima and transition states, with a temperature of 298.15 K. Harmonic frequency calculations were performed for all stationary points, confirming that the points were minima on the surface and exhibited no imaginary frequencies, while transition state structures displayed a single imaginary frequency. Intrinsic reaction coordinate calculations were performed to connect the transition states to the minima in both the forward and reverse directions. We used VMD [45] and CYLView [46] for the graphical representation. The electrostatic potential map for the MD-simulated Michaelis complex and the transition state from the ONIOM (QM/MM) calculation (including only the substrate and the key amino acid residues) were generated using the cubegen tool implemented in Gaussian16.

### MD simulation

Initial atomic coordinates (PDB ID 8QHQ) were taken from the X-ray structure of E104Q-*Hs*DNPH1 bound to 5hmdUMP [6], including crystallographic water, determined at 1.75 Å resolution. MD simulations were carried out on the dimeric protein after replacing Q104 with E using Modeller to restore the WT enzyme. Standard residues were protonated using an H^++^ server [47], where the protonation states of residues were determined at pH 6.0. The resulting charge of −3 was neutralised with 3 Na^+^ counterions. The system was then soaked in a truncated octahedral box of 25774 TIP3P [48] water molecules extending to at least 12 Å from the protein atoms. The ﬀ14SB force ﬁeld [49] described the protein, and the general Amber force field (GAFF2) [50] was performed with Antechamber and parmchk2 for the 5hmdUMP substrate. The charges were derived using the AM1-bcc method [51]. Antechamber generates MM parameters for 5hmdUMP, while parmchk2 verifies this topology and reports missing parameters. The simulation was carried out using the Amber 22 Molecular Dynamics package [52]. The system prepared as described above was ﬁrst subjected to 500 steps of steepest descent and 500 steps of conjugate gradient minimisation, with the protein held ﬁxed by using position restraints with a force constant of 40.0 kcal mol^-1^Å^-2^. An additional 2500 steps of steepest descent and 2500 steps of conjugate gradient minimisation followed. The system was then heated to the target temperature of 300 K for 1 ns under constant volume periodic boundary conditions (NVT), with weak positional restraints applied to the protein atoms (force constant of 10.0 kcal mol^-1^Å^-2^). Subsequently, ~ 8 ns of constant pressure and temperature simulation (NPT) was carried out to equilibrate the system with a force constant of 0.1 kcal mol^-1^Å^-2^. An average pressure of 1 atm was maintained by using isotropic position scaling with a relaxation time of 2 ps. Temperature was controlled via Langevin Dynamics with a collision frequency of 1 ps^-1^. A cut-off of 10 Å was used for non-bonded interactions, and long-range electrostatic interactions were treated using the Particle Mesh Ewald (PME) method [53]. The SHAKE method constrained all bonds involving hydrogen, and the time step for numerical integration was 1 fs. The last 1 ns was then used to obtain the average geometry of the protein and the substrate. The simulation results were analysed using the ptraj program in the Amber 22 package [52] and VMD.

## Supplementary material

online supplementary figure 1.

## Data Availability

All data supporting the work are included in the main article and supplementary information file. Files and data underpinning the computational models of ground and transition states have been deposited to FigShare and are available at https://figshare.com/s/00c42649f0dbad15d99c [54]. Additional information is available from the corresponding author upon request.

## Open Access

This article has been published open access under our Subscribe to Open programme, made possible through the support of our subscribing institutions, learn more here: https://portlandpress.com/pages/open_access_options_and_prices#conditional

## Abbreviations

*Hs*DNPH1: human enzyme 2′-deoxynucleoside 5′-phosphate N-hydrolase 1
ITC: isothermal titration calorimetry
RMSD: root mean square deviation
5hmdUMP: 5-hydroxymethyl-2′-deoxyuridine 5′-monophosphate
