# Supplementary material for: The energy landscape of N-ribosidic bond cleavage catalysed by 2′-deoxynucleoside 5′-phosphate N-hydrolase 1
Source: Biochem J. 2025 Dec 17;482(24):1897–918. doi: 10.1042/BCJ20253400 (PMC12751057; doi:10.1042/BCJ20253400)
Supplement: online supplementary figure 1. [file bcj-482-24-BCJ20253400-s001.pdf]

Supplementary Information

The energy landscape of *N*-ribosidic bond cleavage catalysed by 2′-deoxynucleoside 5′-phosphate *N*-hydrolase 1

Anna E. Carberry,[1] Tamal Das,[2] David J. Harrison,[3,4] Jennifer S. Hirschi,[2,*] and Rafael G. da Silva[1,*]

[1]School of Biology, Biomedical Sciences Research Complex, University of St Andrews, St Andrews, KY16 9ST, United Kingdom

[2]Department of Chemistry, Binghamton University, Vestal, NY 13850, United States

[3]School of Medicine, University of St Andrews, St Andrews, KY16 9TF, United Kingdom

[4]NuCana plc, Edinburgh, EH12 9DT, United Kingdom

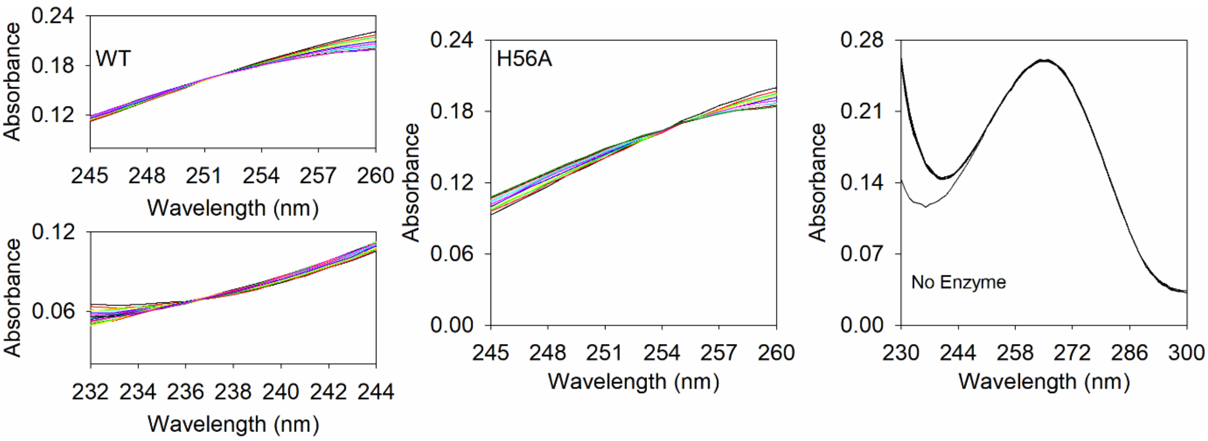

Supplementary Figure S1. Close-up view of isosbestic points in the time-dependent UV absorbance spectra of the reaction catalysed by *Hs*DNPH1 variants, and time-dependent UV absorbance control spectra of the substrate 5hmdUMP in the absence of enzyme. In the control spectra, the grey line is the spectrum at time = 0, whereas the black line is the spectrum at time = 2 h. The region in the spectra between 230 nm and 244 nm was not included in the reaction analysis for H56A-*Hs*DNPH1 because it also changes in the control lacking enzyme over the longer timescale needed with this variant.

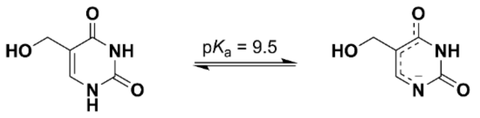

Supplementary Figure S2. Acid-base equilibrium between neutral and anionic 5hmUra.

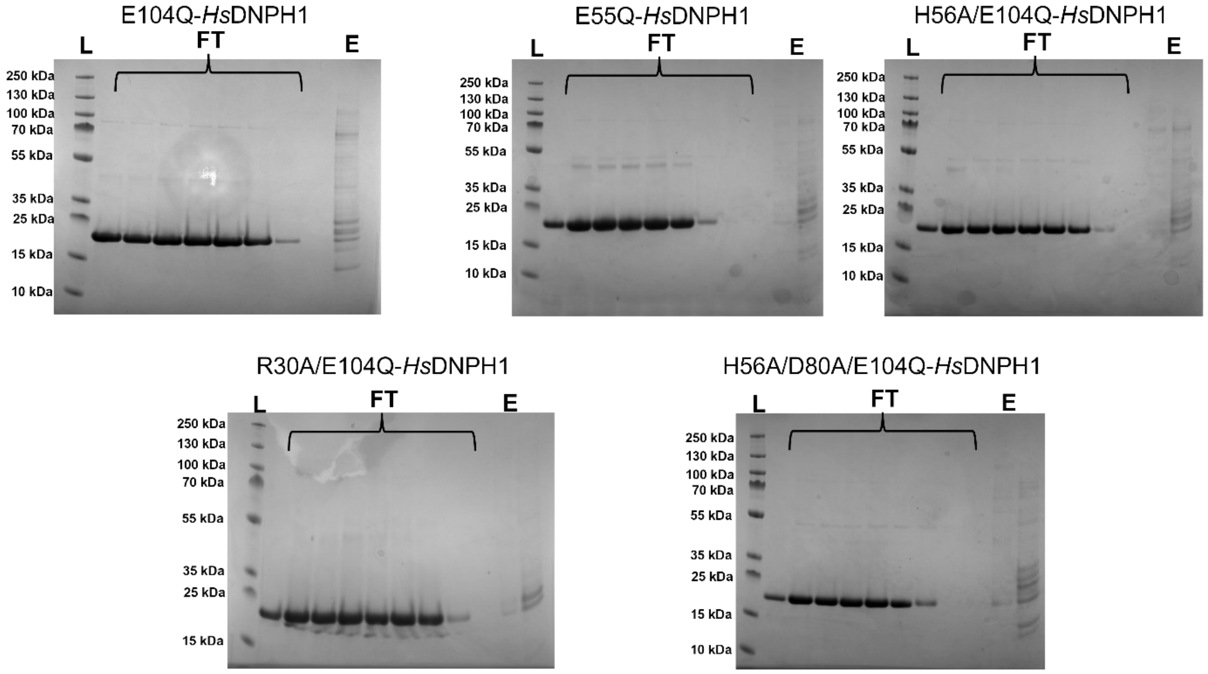

Supplementary Figure S3. SDS-PAGE analysis of purified *Hs*DNPH1 variants. Lanes are as follows: L is the MW marker (PageRuler Plus Prestained Protein Ladder); FT is the flowthrough from the second chromatography, which was pooled; E is the elution, which was discarded.

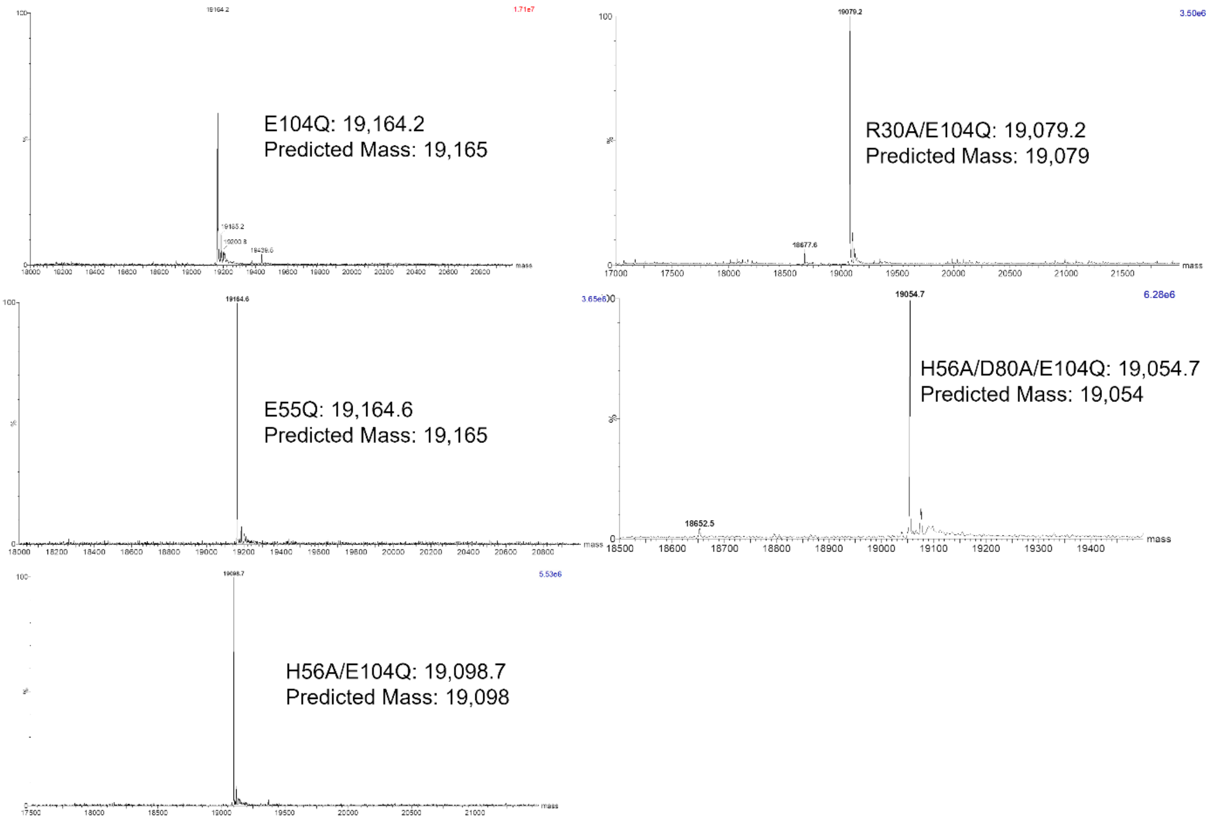

Supplementary Figure S4. ESI-TOF-MS analysis of *Hs*DNPH1 variants.

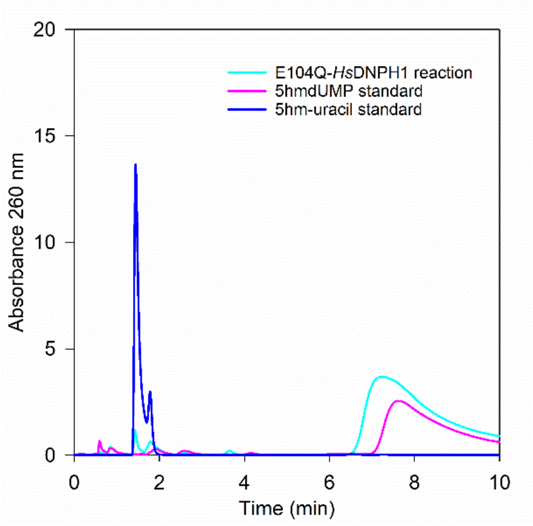

Supplementary Figure S5. HPLC elution profiles with E104Q-*Hs*DNPH1. The substrate 5hmdUMP elutes at ~6.9 min, whereas the product 5hmUra elutes at ~1.5 min.

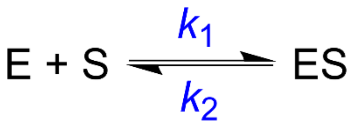

Supplementary Figure S6. Single-step binding model between enzyme and substrate.

Supplementary Table S1. Rate and equilibrium constants governing binding of *Hs*DNPH1 variants to 5hmdUMP.[a]

Constant	E104Q-*Hs*DNPH1	H56A/E104Q-*Hs*DNPH1
k_1 (μM^{-1} s^{-1})	1.253 ± 0.007<br>(1.05 – 1.53)	0.997 ± 0.003<br>(0.91 – 1.1)
k_2 (s^{-1})	4.39 ± 0.07<br>(2.62 – 6.85)	16.95 ± 0.07<br>(14.9 – 19.3)
K_D^{app} (μM)	3.50 ± 0.06	17.00 ± 0.09
**Experiment**	Binding kinetics and simultaneous numerical integration of data at 251 nm and 286 nm	Binding kinetics and numerical integration of data at 286 nm

[a]Data are mean ± best-fit uncertainty from numerical integration. Data in brackets are FitSpace boundaries.

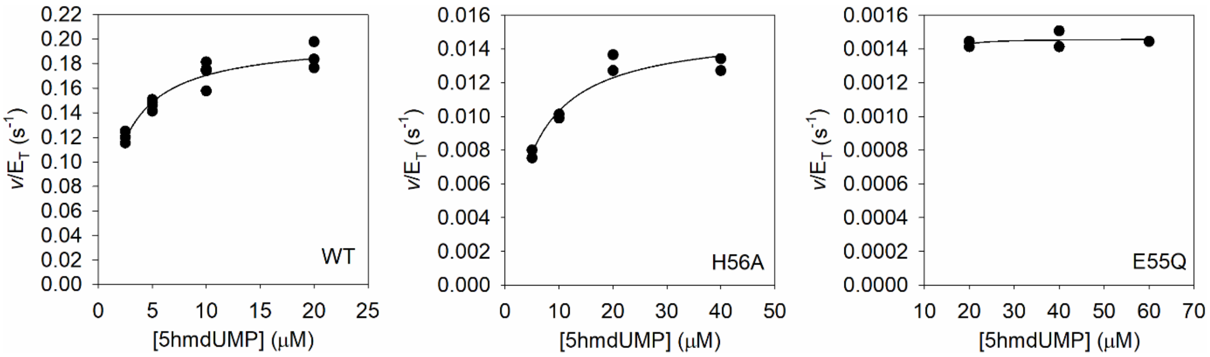

Supplementary Figure S7. Substrate saturation curves for *Hs*DNPH1 variants assayed at 275 nm. All data points are shown for two independent measurements, except for WT-*Hs*DNPH1 where four independent measurements were carried out. Lines are best fit of the data to either equation 1 (WT- and H56A-*Hs*DNPH1) or equation 3 (E55Q-*Hs*DNPH1).

Supplementary Table S2. Rate and equilibrium constants governing the steady-state and single-turnover reactions of *Hs*DNPH1 variants with 5hmdUMP.

**Constant**	***Hs*DNPH1**			
	**WT- (pH 7.0)**	**WT- (pH 8.5)**	**H56A-**	**E55Q-**
k_{cat} (s^{-1})[a]	0.210 ± 0.001	0.054 ± 0.002	0.015 ± 0.001	0.0015 ± 0.0001
k_{cat}/K_M ($M^{-1}s^{-1}$)	≥ 8.4 × 10^4	≥ 1.1 × 10^4	≥ 3,040	Not estimated
k_1 (μM^{-1} s^{-1})[b]	1.037 ± 0.003<br>(0.92 – 1.19)	0.204 ± 0.001<br>(0.163 – 0.255)	0.520 ± 0.002<br>(0.466 – 0.582)	1.32 ± 0.01<br>(1.14 – 1.55)
k_2 (s^{-1})[b]	2.45 ± 0.02<br>(1.44 – 3.87)	1.01 ± 0.02<br>(0.33 – 2.12)	3.61 ± 0.03<br>(3.02 – 4.31)	5.88 ± 0.08<br>(3.93 – 8.62)
K_D^{app} (μM)[b]	2.36 ± 0.02	5.0 ± 0.1	6.94 ± 0.06	4.45 ± 0.07
k_3 (s^{-1})[b]	0.219 ± 0.001<br>(0.2 – 0.24)	0.353 ± 0.001<br>(0.313 – 0.393)	0.01787 ± 0.00008<br>(0.017 – 0.019)	0.271 ± 0.001<br>(0.251 – 0.292)
**Experiment**	Steady state at 275 nm; and simultaneous numerical integration of STO data at 251 nm and 286 nm	Steady state at 275 nm; and numerical integration of STO data at 286 nm	Steady state at 275 nm; and numerical integration of STO data at 286 nm	Steady state at 275 nm; and numerical integration of STO data at 286 nm

[a]Data are fitting value ± best fit error from analytical fitting. [b]Data are mean ± best-fit uncertainty from numerical integration. Data in brackets are FitSpace boundaries.

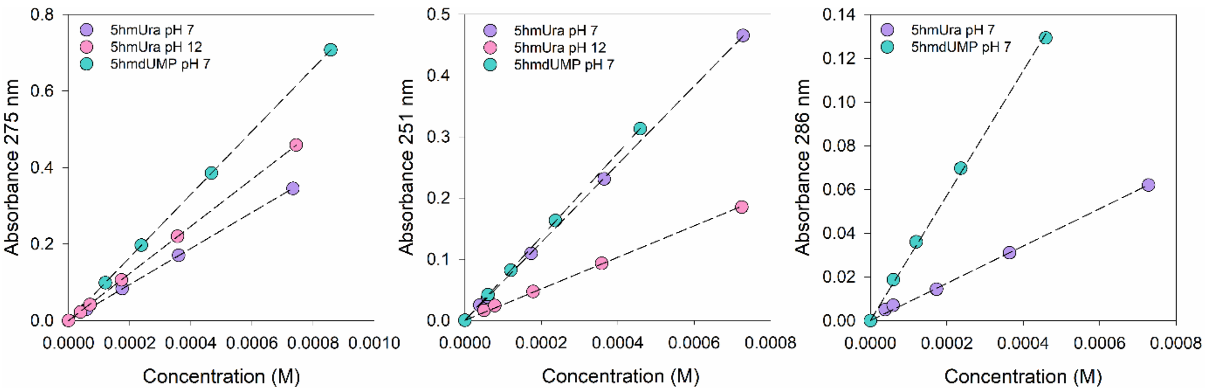

Supplementary Figure S8. Determination of the extinction coefficients for 5hmdUMP and 5hmUra at different wavelengths and pHs. $\varepsilon_{275\ nm}$ for 5hmUra is 4,712 ± 12 M^{-1} cm^{-1} at pH 7 and 6,161 ± 13 M^{-1} cm^{-1} at pH 12, and for 5hmdUMP, 8,251 ± 4 M^{-1} cm^{-1}. $\varepsilon_{251\ nm}$ for 5hmUra is 6,379 ± 11 M^{-1} cm^{-1} at pH 7 and 2,572 ± 30M^{-1} cm^{-1} at pH 12, and for 5hmdUMP, 6,836 ± 15M^{-1} cm^{-1}. $\varepsilon_{286\ nm}$ for 5hmUra is 853 ± 14 M^{-1} cm^{-1} and for 5hmdUMP, 2,856 ± 33 M^{-1} cm^{-1}, at pH 7.0. All data points are shown for three independent measurements. Dashed lines are linear regressions of the data.

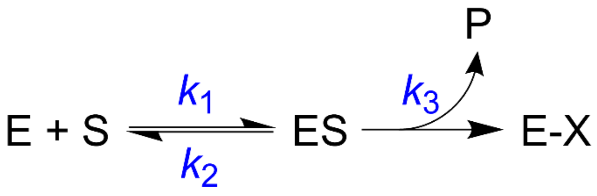

Supplementary Figure S9. The first half-reaction model. It encompasses single-step substrate binding and catalysis to release the first product and form the enzyme-intermediate adduct.

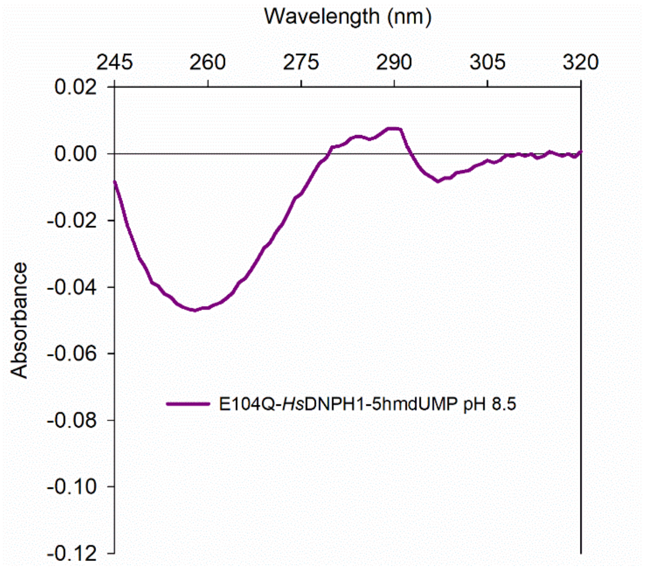

Supplementary Figure S10. Difference UV spectrum of 5hmdUMP bound to WT-*Hs*DNPH1 at pH 8.5.

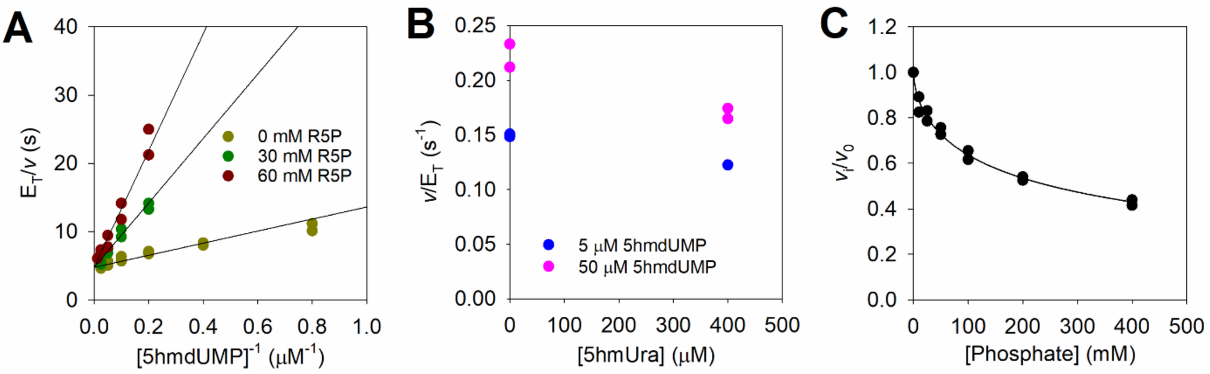

Supplementary Figure S11. WT-*Hs*DNPH1 inhibition studies. **(A)** WT-*Hs*DNPH1 inhibition by ribose 5-phosphate (R5P). Lines are the double-reciprocal of the best fit of the data to equation 4. **(B)** WT-*Hs*DNPH1 inhibition by 5hmUra. The maximum concentration of inhibitor was 400 μM becasue beyond this concentration, 5hmUra absorbance at 275 nm interfered with the assay. **(C)** WT-*Hs*DNPH1 inhibition by phosphate. The line is best fit of the data to equation 5. In all cases, all data points are shown for two independent measurements.

Supplementary Table S3. Simultaneous fit of the single- and multiple-turnover reactions of *Hs*DNPH1 variants with 5hmdUMP.[a]

**Constant**	***Hs*DNPH1**	
	**WT- (pH 8.5)**	**E55Q- (pH 7.0)**
k_1 **(μM^{-1}s^{-1})**	0.199 ± 0.0009<br>(0.168 – 0.236)	1.309 ± 0.006<br>(1.12 – 1.55)
k_2 **(s^{-1})**	1.05 ± 0.02<br>(0.47 – 1.88)	5.69 ± 0.07<br>(3.62 – 8.5)
K_D^{app} **(μM)**	5.3 ± 0.1	4.35 ± 0.06
k_3 **(s^{-1})**	0.372 ± 0.0008<br>(0.343 – 0.403)	0.2807 ± 0.0005<br>(0.264 – 0.299)
k_5 **(s^{-1})**	0.0894 ± 0.0003<br>(0.0803 – 0.0993)	0.00159 ± 0.00004<br>(0.00213 – 0.00292)

[a]Data are mean ± best-fit uncertainty from numerical integration. Data in brackets are FitSpace boundaries.

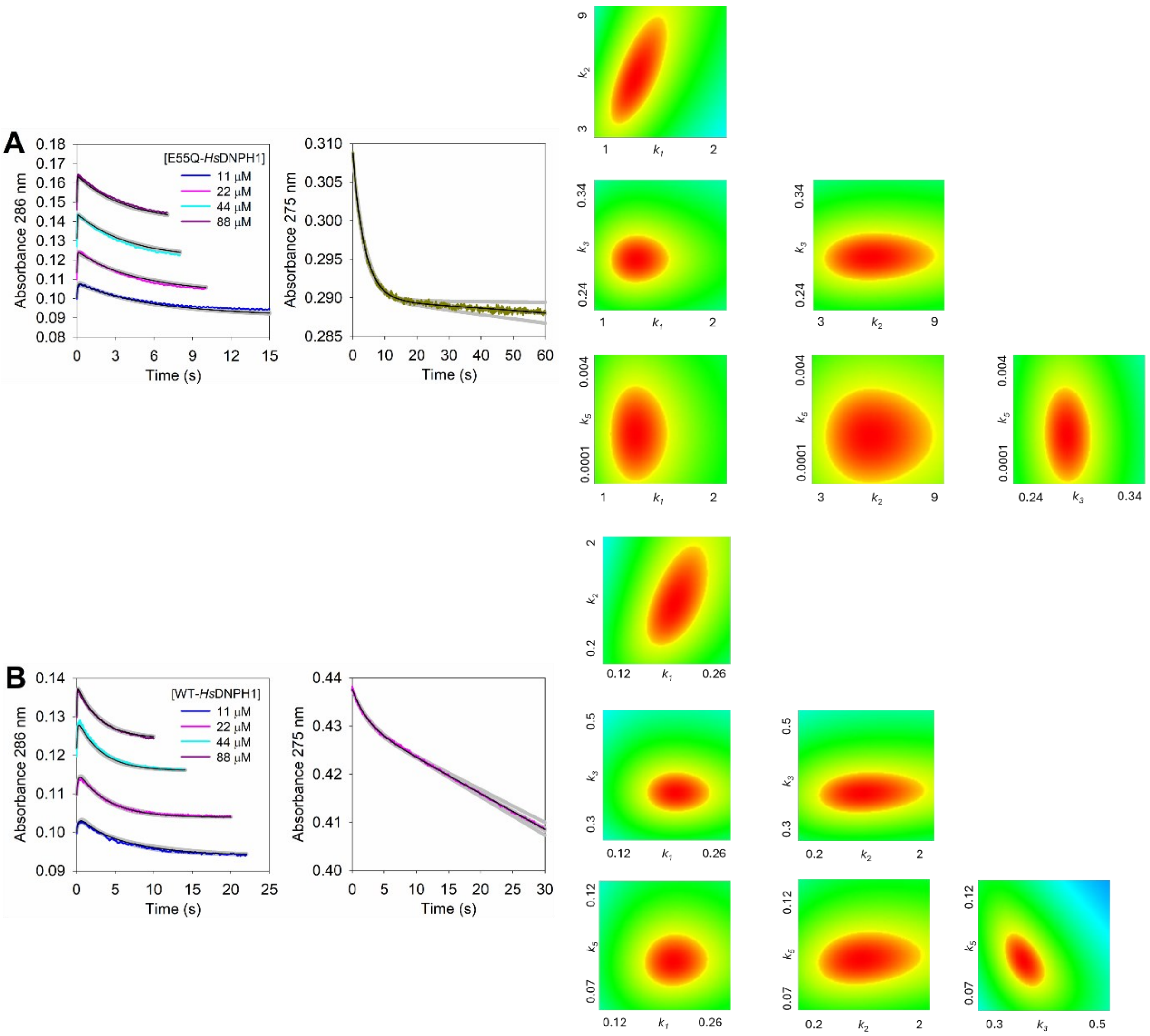

Supplementary Figure S12. Simultaneous fit of single- and multiple-turnover rapid kinetics data. **(A)** E55Q-*Hs*DNPH1 reaction with 5hmdUMP and FitSpace contour plot of the best-fit model relative to the data. **(B)** WT-*Hs*DNPH1 reaction with 5hmdUMP at pH 8.5 and FitSpace contour plot of the best-fit model relative to the data. Lines in colour are experimental data, black lines are numerical integration-based fit to a three-step reaction model, and grey lines are boundaries produced by FitSpace analysis. In the contour plots, constrained boundaries are defined by regions in red; k_1 is in units of μM^{-1} s^{-1}, and k_2, k_3 and k_5 in s^{-1}.

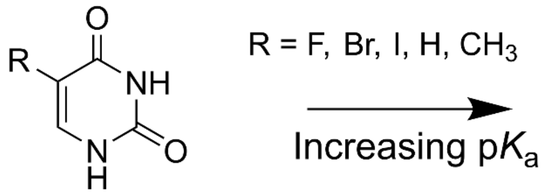

Supplementary Figure S13. Leaving group structures used for the linear free energy relationships.

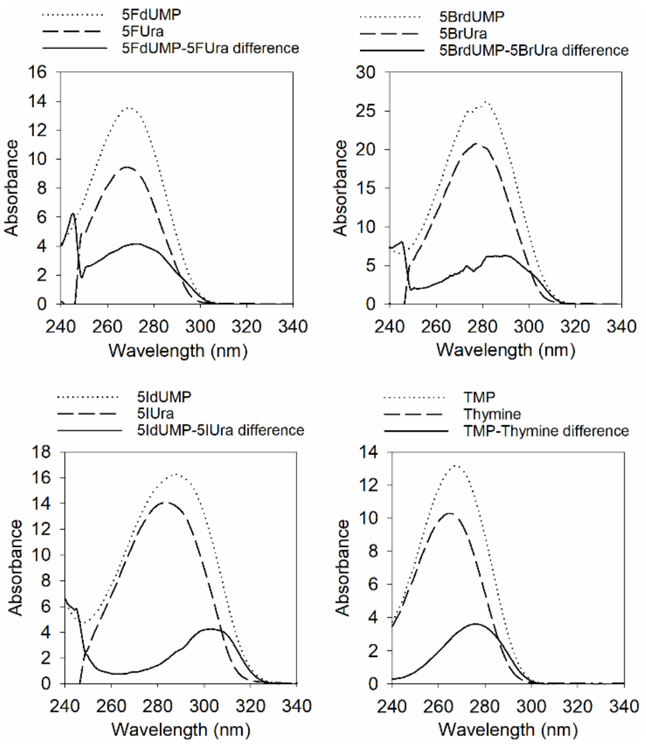

Supplementary Figure S14. UV-VIS spectra of substrates and products used in the linear free energy relationship studies.

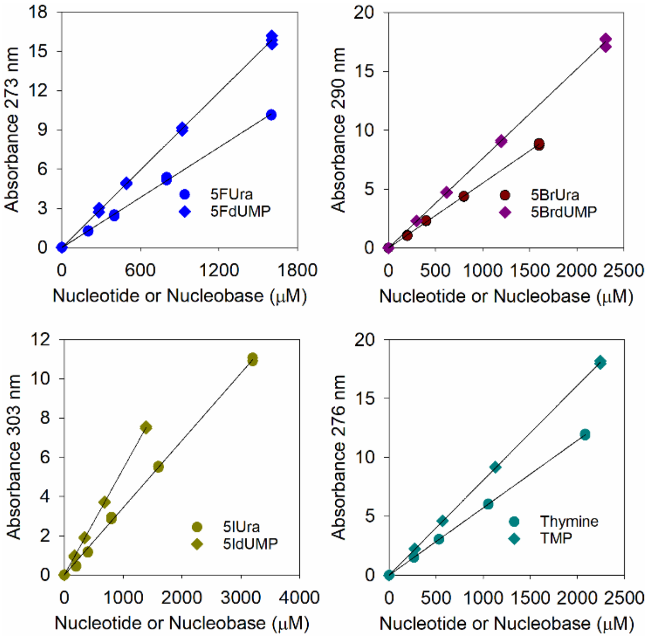

Supplementary Figure S15. Determination of the extinction coefficients for nucleotides and nucleobases relevant for the linear free energy relationship analyses. $\varepsilon_{273\ nm}$ for 5FUra is 6,400 ± 39 M^{-1} cm^{-1} and for 5FdUMP, 9,900 ± 48 M^{-1} cm^{-1}. $\varepsilon_{290\ nm}$ for 5BrUra is 5,500 ± 22 M^{-1} cm^{-1} and for 5BrdUMP, 7,600 ± 34 M^{-1} cm^{-1}. $\varepsilon_{303\ nm}$ for 5IUra is 3,400 ± 25 M^{-1} cm^{-1} and for 5IdUMP, 5,400 ± 12 M^{-1} cm^{-1}. $\varepsilon_{276\ nm}$ for thymine is 5,700 ± 6 M^{-1} cm^{-1} and for 5IdUMP, 8,100 ± 15 M^{-1} cm^{-1}. All data points are shown for three independent measurements. Lines are linear regressions of the data.

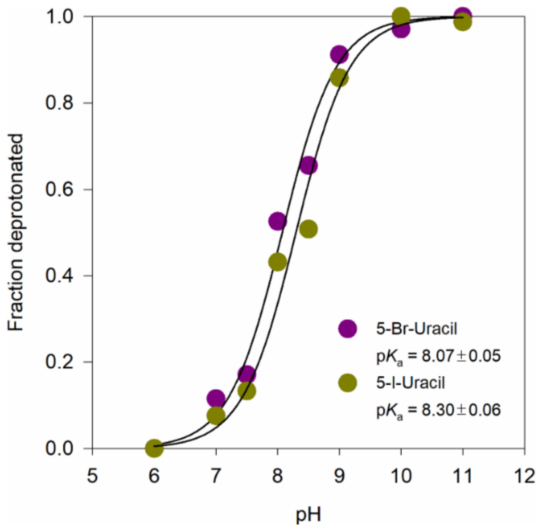

Supplementary Figure S16. Determination of the pK_a of 5-Br-uracil and 5-I-uracil, based on normalised absorbance at 300 nm and 305 nm, respectively. The absorbance of the nucleobases those respective wavelengths increases with increasing pH. Data are mean ± standard error from three independent measurements. The lines are best fit of the data to the normalised Henderson-Hasselbalch equation.

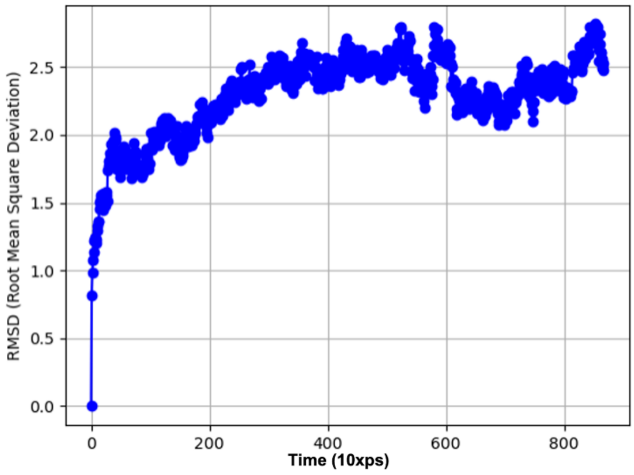

Supplementary Figure S17. Time evolution of the RMSD of the *Hs*DNPH1-5hmdUMP complex.

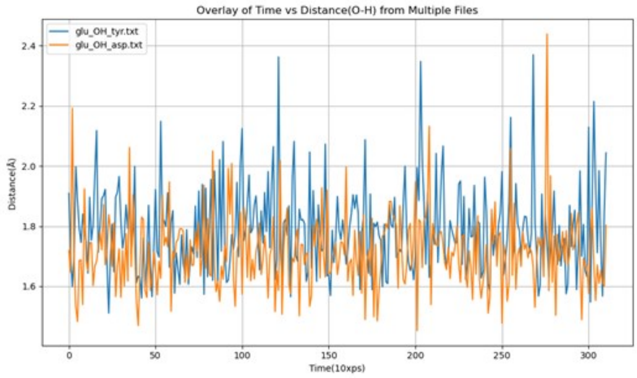

Supplementary Figure S18. Fluctuation of the O --- H distance in the H-bonds between E104 and Y24 (blue) as well as E104 and D80 (orange) during the equilibration of 5hmdUMP-bound *Hs*DNPH1.

Supplementary Table S4. Oligonucleotide primers used for site-directed mutagenesis.

Mutation	Forward Primer	Reverse Primer
**H56A**	5′-CCGAAGCTGTGGCTGCTGCTGAGCTGGGGGCC-3′	5′-CAGCCACAGCTTCGGTTAACACGGTTCCAAAACGGC-3′
**R30A**	5′-GCATTGCTGGTGGACGTGAGGACCGCACCCTGTAC-3′	5′-CGTCCACCAGCAATGCTTCCGCAGAAATAAAGTGCCG-3′
**E104Q**	5′-GGTTATCAACTGGGACGCGCCGTGGCATTCAATAAAC-3′	5′-CGTCCCAGTTGATAACCGACACCAAGGGAAGGTTGAG-3′
**E55Q**	5′-GTTAACCCAACATGTGGCTGCTGCTGAGCTGGG-3′	5′-CACATGTTGGGTTAACACGGTTCCAAAACGGCGC-3′
**D80A**	5′-CATGAGCAAGCTTTGGAGTGGTTACAACAGGCGG-3′	5′-CCAAAGCTTGCTCATGGATAAGGCGGTCTCCTCC-3′
